# Physiological Network From Anthropometric and Blood Test Biomarkers

**DOI:** 10.3389/fphys.2020.612598

**Published:** 2021-01-12

**Authors:** Antonio Barajas-Martínez, Elizabeth Ibarra-Coronado, Martha Patricia Sierra-Vargas, Ivette Cruz-Bautista, Paloma Almeda-Valdes, Carlos A. Aguilar-Salinas, Ruben Fossion, Christopher R. Stephens, Claudia Vargas-Domínguez, Octavio Gamaliel Atzatzi-Aguilar, Yazmín Debray-García, Rogelio García-Torrentera, Karen Bobadilla, María Augusta Naranjo Meneses, Dulce Abril Mena Orozco, César Ernesto Lam-Chung, Vania Martínez Garcés, Octavio A. Lecona, Arlex O. Marín-García, Alejandro Frank, Ana Leonor Rivera

**Affiliations:** ^1^Posgrado en Ciencias Biomédicas, Facultad de Medicina, Universidad Nacional Autónoma de México, Ciudad de México, Mexico; ^2^Centro de Ciencias de la Complejidad, Universidad Nacional Autónoma de México, Ciudad de México, Mexico; ^3^Instituto de Ciencias Nucleares, Universidad Nacional Autónoma de México, Ciudad de México, Mexico; ^4^Subdirección de Investigación Clínica, Instituto Nacional de Enfermedades Respiratorias, Ciudad de México, Mexico; ^5^Facultad Mexicana de Medicina, Universidad La Salle, Ciudad de México, Mexico; ^6^Unidad de Investigación en Enfermedades Metabólicas, Instituto Nacional de Ciencias Médicas y Nutrición Salvador Zubirán, Ciudad de México, Mexico; ^7^Tecnológico de Monterrey, Escuela de Medicina y Ciencias de la Salud, Monterrey, Mexico; ^8^Departamento de Investigación en Inmunología y Medicina Ambiental, Instituto Nacional de Enfermedades Respiratorias, Ciudad de México, Mexico; ^9^Cátedras CONACYT, Ciudad de México, Mexico; ^10^Unidad de Urgencias Respiratorias, Instituto Nacional de Enfermedades Respiratorias, Ciudad de México, Mexico; ^11^Programa de Estudios Combinados en Medicina, Facultad de Medicina, Universidad Nacional Autónoma de México, Ciudad de México, Mexico; ^12^El Colegio Nacional, Ciudad de México, Mexico

**Keywords:** physiological networks, complex inference network, homeostasis, anthropometric measures, blood test biomarkers

## Abstract

Currently, research in physiology focuses on molecular mechanisms underlying the functioning of living organisms. Reductionist strategies are used to decompose systems into their components and to measure changes of physiological variables between experimental conditions. However, how these isolated physiological variables translate into the emergence -and collapse- of biological functions of the organism as a whole is often a less tractable question. To generate a useful representation of physiology as a system, known and unknown interactions between heterogeneous physiological components must be taken into account. In this work we use a Complex Inference Networks approach to build physiological networks from biomarkers. We employ two unrelated databases to generate Spearman correlation matrices of 81 and 54 physiological variables, respectively, including endocrine, mechanic, biochemical, anthropometric, physiological, and cellular variables. From these correlation matrices we generated physiological networks by selecting a *p*-value threshold indicating statistically significant links. We compared the networks from both samples to show which features are robust and representative for physiology in health. We found that although network topology is sensitive to the *p*-value threshold, an optimal value may be defined by combining criteria of stability of topological features and network connectedness. Unsupervised community detection algorithms allowed to obtain functional clusters that correlate well with current medical knowledge. Finally, we describe the topology of the physiological networks, which lie between random and ordered structural features, and may reflect system robustness and adaptability. Modularity of physiological networks allows to explore functional clusters that are consistent even when considering different physiological variables. Altogether Complex Inference Networks from biomarkers provide an efficient implementation of a systems biology approach that is visually understandable and robust. We hypothesize that physiological networks allow to translate concepts such as homeostasis into quantifiable properties of biological systems useful for determination and quantification of health and disease.

## Introduction

Communication and interaction between physiological systems and organs are the essence of physiology (Ganong, 1969; Bashan et al., 2012; Bartsch et al., 2015; Ivanov et al., 2016). This integration of organisms as a whole results in an inherent complexity of physiological phenomena (Burggren et al., 2005) that has implications for the behavior of physiological systems in health and disease. For example, it has become clear that the simultaneous occurrence of diseases in the same individual (comorbidity) occurs more often than would be expected from the individual prevalence of each disease by chance alone (Alberti et al., 2009). Additionally, when a comorbid state is present, the clinical expression of each individual disease is usually more difficult to treat and associated with worsened outcomes (Wu et al., 2019). Although these observations are common in medical practice, relatively few health conditions are regarded with an extensive perspective. Some of the most common examples are the metabolic syndrome (Alberti et al., 2009), and the asthma-obesity-diabetes triad (Wu et al., 2019). Mainstream methodology in disease diagnosis and disease treatment employs a reductionist approach to physiology, where at most two variables are studied simultaneously. This presents an immediate challenge for the study of complex comorbidities where several physiological systems are involved. An emerging paradigm for this problem is the systems biology perspective, where the organism is visualized as an open system composed of interacting components (Von Bertalanffy, 1968). The integration of these body components generates physiological states that can be studied in health and disease through complexity approaches (Ivanov et al., 2016). A way of representing and conceptualizing systems is through networks. This approach facilitates the visualization and analysis of potentially large numbers of interactions (Pavlopoulos et al., 2011). Networks have been applied in very diverse fields of science, including economy, sociology, ecology, and they have been generalized recently for the study of biomedical sciences (Albert and Barabási, 2002; Boccaletti et al., 2006). Currently, most approaches to network analysis in biomedical science are restricted to homogeneous datasets, i.e., where all the variables and interactions are of the same kind, e.g., differential gene expression networks. However, physiology is not constructed from interactions between components that are all of the same kind. Some novel approaches to address physiological networks have been developed where physiological integration of different systems within the organism is demonstrated through time-series analysis (Ivanov and Bartsch, 2014). Multiple time scales may be involved in different physiological interactions and their measurements (Bartsch et al., 2014). For instance, some physiological interactions occur in seconds and are measured with great accuracy in fractions of seconds. Other physiological interactions occur in cycles of days or months and may only be quantified as isolated point measurements and not continuously as time series (Barajas-Martínez et al., 2020). Moreover, while networks are usually constructed through links that are associated with known, experimentally verified interactions, such as the Kyoto Encyclopedia of Genes and Genomes, KEGG (Ogata et al., 1999), it is likely that certain important interactions in biological systems remain unknown. The methodology of Complex Inference Networks allows the construction of networks where the links are inferred, instead of being directly observed. Correlation networks are a common and widespread method to make such inferences (Batushansky et al., 2016) that may be later verified through conventional mechanistic studies. A network approach provides also a new level of study where global properties of the system, that are not apparent at the local level, emerge from the interactions of the multiple components. These interactions are revealed by changes in topology and connectivity (Ivanov and Bartsch, 2014).

How to approach multivariate datasets to generate insight in physiology is an area under development. Principal Component Analysis (PCA) and network analysis are two current data analysis techniques that have been transferred to biology from other areas of science (Liu et al., 2015; Asada et al., 2016). The coupling between physiological variables can be explored through the change in the covariance observed in different samples (Hofer and Sliwinski, 2001). For correlation networks, an association would be found between those variables that interact directly or indirectly within the physiological network. Here, the physiological network is modeled as a continuous association of pairs of variables. For this correlation model, a correlation matrix was constructed for the chosen physiological parameters. These variables are often of different nature, ions in solution, mechanical forces and hormones. Their interactions are also of different kinds, direct and indirect, through very different physiological mechanisms. In summary, physiological variables are correlated along all their biologically plausible spectrum. In this scenario, the associations between parameters are present even for healthy values and represent a continuum. From the systems biology perspective, the network structure is a direct result of the coordination, or lack thereof, of components that are linked by homeostatic feedback (Goldstein, 2019). For example, in a simple negative feedback a change in a regulated variable is detected by a comparator in the organism that through effector variables counteracts the perturbation (Fossion et al., 2018). These variables, along with buffer variables, result in the covariance of multiple variables in biological systems.

The aim of this work is to generate a mainstream workflow for developing physiological networks from heterogeneous datasets including endocrine, mechanic, biochemical, anthropometric, vital signs, and cellular elements that are readily accessible and already being employed without a holistic perspective. In this contribution we have constructed a physiological network for control subjects (young adults, asymptomatic, clinically diagnosed as healthy) from physiological, biochemical, and anthropometric data.

## Methodology

### Ethics Statement

The study was developed according to Good Clinical Practice guidelines and the Declaration of Helsinki. All procedures involving participants were in accordance with these ethical standards and followed the procedures required by the corresponding ethics committees. All the participants signed a written informed consent form with full knowledge of the interventions involved in this protocol. All databases employed here were constructed with authorization of the Ethics Committees as detailed below.

### Databases

In the present contribution, 2 different datasets of multivariate and heterogeneous physiological data were analyzed (**C22_14** and **Project_42**) allowing to compare the physiological networks obtained from different datasets and to confirm the robustness of the approach and the consistency of the results obtained. The **C22_14** database comprised 81 variables of which 46 were unique; the **Project_42** database recorded 55 variables of which 19 were unique; 36 variables were in common between both datasets (see Table 1). The physiological network corresponding to the 36 variables in common was also constructed to allow comparison between both datasets.

**TABLE 1:** Description of physiological variables.

**Category**	**ID**	**database**	**Name**	**Variable**	**Description**	**Units**
Vital signs	1	C22_14 Project_42	SBP	Systolic blood pressure	Pressure of the blood in the arteries when the heart pumps. It is the higher of two blood pressure measurements.	mmHg
	2	C22_14 Project_42	DBP	Diastolic blood pressure	Pressure of the blood in the arteries when the heart is filling. It is the lower of two blood pressure measurements.	mmHg
Anthropometric measures	3	C22_14 Project_42	waist	Waist circumference	Waist size (waist circumference) is an indicator of abdominal obesity.	cm
	4	C22_14 Project_42	Wt	Weight	Amount that someone weighs.	kg
	5	C22_14 Project_42	Ht	Height	Anthropometric measurement of size (length from the bottom to the top).	cm
Bioimpedance	6	C22_14 Project_42	BF	Body fat	Estimated amount of fat weight through bioimpedance.	kg
	7	C22_14 Project_42	SMM	Skeletal muscle mass	Estimated weight of muscle.	kg
	8	C22_14 Project_42	TBW	Total body water	Estimated amount of water through bioimpedance.	kg
	9	C22_14 Project_42	VF	Visceral fat	High risk adiposity surrounding internal organs.	kg
Hematic biometry	10	C22_14 Project_42	Leuk	Leukocytes	White blood cells concentration.	10^3^/mm^3^
	11	C22_14 Project_42	Neut	Neutrophiles	Innate immunity white blood cell.	10^3^/mm^3^
	12	C22_14 Project_42	Lymph	Lymphocytes	Adaptative immunity white blood cell.	10^3^/mm^3^
	13	C22_14 Project_42	Mono	Monocytes	Innate immunity macrophage precursor.	10^3^/mm^3^
	14	C22_14 Project_42	Eos	Eosinophiles	Allergic and parasitic response blood cell.	10^3^/mm^3^
	15	C22_14 Project_42	Baso	Basophiles	Least common type of granulocyte.	10^3^/mm^3^
	16	C22_14 Project_42	Eryt	Erythrocytes	Oxygen transport blood cell.	10^6^/mm^3^
	17	C22_14 Project_42	Hb	Hemoglobin	Oxygen carrier protein.	g/dL
	18	C22_14 Project_42	Hto	Hematocrit	Percentage of total blood.	%
	19	C22_14 Project_42	MCV	Mean corpuscular volume	Erythrocyte volume.	fL
	20	C22_14 Project_42	MCH	Mean corpuscular hemoglobin	Erythrocyte hemoglobin amount.	Pg
	21	C22_14 Project_42	MCHC	Mean corpuscular hemoglobin concentration	Erythrocyte hemoglobin concentration.	g/dL
	22	C22_14 Project_42	RDW	Red cell distribution width	Erythrocyte distribution amplitude.	%
	23	C22_14 Project_42	Plat	Platelets	Cell fragments involved in coagulation.	10^3^/mm^3^
	24	C22_14 Project_42	MPV	Mean platelet volume	Platelet volume.	fL
Blood chemistry	25	C22_14 Project_42	Alb	Albumin	Hepatic protein in blood responsible for oncotic pressure.	g/dL
	26	C22_14 Project_42	TGlyc	Triglycerides	Group of lipids, glycerol ester with three fatty acids, for transport.	mg/dL
	27	C22_14 Project_42	Chol	Total cholesterol	Total cholesterol amount regardless of the fraction.	mg/dL
	28	C22_14 Project_42	HDL	High density lipoprotein cholesterol	Lipoprotein carrier from cells to lipid depots.	mg/dL
	29	C22_14 Project_42	LDL	Low density lipoprotein cholesterol	Lipoprotein carrier from lipid depots to cells.	mg/dL
	30	C22_14 Project_42	Gluc	Glucose	Carbohydrate, main cellular energy substrate.	mg/dL
	31	C22_14 Project_42	Urea	Urea	Aminoacidic degradation end product.	mg/dL
	32	C22_14 Project_42	Uric	Uric Acid	Purine degradation final metabolite for excretion.	mg/dL
	33	C22_14 Project_42	Creat	Creatinine	Creatine muscle waste product with constant excretion rate.	mg/dL
	34	C22_14 Project_42	HbA1c	Glycated hemoglobin	A1c fraction of glycated hemoglobin.	%
Molecular biology	35	C22_14 Project_42	CRP	C reactive protein	Acute phase pentraxin, unspecific biomarker of inflammation.	mg/dL
	36	C22_14 Project_42	Ins	Insulin	Single hypoglycemic hormone produced by beta cells.	pg/ml
Blood chemistry	37	Project 42	Cl	Serum chlorine	Serum electrolyte concentrations are the net result of intake, excretion, and shifts between intra- and extracellular fluids.	mEq/L
	38	Project 42	K	Serum potassium	Serum electrolyte concentrations are the net result of intake, excretion, and shifts between intra- and extracellular fluids.	mEq/L
	39	Project 42	Na	Serum sodium	Serum electrolyte concentrations are the net result of intake, excretion, and shifts between intra- and extracellular fluids.	mEq/L
	40	Project 42	Bl_T	Total bilirrubin	Hemoglobin degradation product.	mg/dL
	41	Project 42	Bl_D	Direct bilirrubin	Unconjugated bilirubin is formed by the breakdown of hemoglobin in the red blood cells.	mg/dL
	42	Project 42	Bl_I	Indirect bilirrubin	Conjugated bilirrubin is produced in the liver for excretion.	mg/dL
	43	Project 42	AST	Aspartate aminotransferase	Hepatic enzyme released in liver injury.	U/L
	44	Project 42	Ca	Serum calcium	Serum electrolyte concentrations are the net result of intake, excretion, and shifts between intra- and extracellular fluids.	mEq/L
	45	Project 42	P	Serum phosphorus	Serum electrolyte concentrations are the net result of intake, excretion, and shifts between intra- and extracellular fluids.	mEq/L
Anthropometric measures	46	Project 42	Tric	Tricipital skinfold	Skinfold thickness measured for the estimation of body fat by measurement of subcutaneous adipose tissue.	mm
	47	Project 42	Bici	Bibipital skinfold	Skinfold thickness measured for the estimation of body fat by measurement of subcutaneous adipose tissue.	mm
	48	Project 42	SupI	Suprailiac skinfold	Skinfold thickness measured for the estimation of body fat by measurement of subcutaneous adipose tissue.	mm
	49	Project 42	SubE	Subescapular skinfold	Skinfold thickness measured for the estimation of body fat by measurement of subcutaneous adipose tissue.	mm
	50	Project 42	Arm	Arm diameter	Anthropometric measurement employed for assessment of cardiovascular risk for identification of central obesity.	cm
	51	Project 42	Hip	Hip circumference	Anthropometric measurement employed for nutritional status evaluation.	cm
Vital signs	52	Project 42	AxilarT	Axilar temperature	Arm-pit temperature measured using a mercury thermometer that reflects body core temperature near the axillary artery.	°C
	53	Project 42	EarT	Auricular temperature	Tympanic temperature reflects body core temperature as it shares arterial blood supply from carotid artery.	°C
	54	Project 42	WristT	Wrist temperature	Peripheral temperature measured at the wrist.	°C
Hematic biometry	55	Project 42	Segm	Segmented neutrophils		103/mm3
Anthropometric measures	56	C22_14	neck	Neck circumference	Neck circumference is a screening measure for identifying overweight and obesity, it reflects upper-body fat distribution and central obesity.	cm
Bioimpedance	57	C22_14	SF	Superficial fat	Low risk adiposity in subcutaneous tissues.	kg
	58	C22_14	Rest	Resting metabolic rate	Energy expenditure at rest.	kcal/day
	59	C22_14	Lean	Lean mass	Estimated amount of lean mass weight through bioimpedance.	kg
	60	C22_14	ECW	Extracellular water	Amount of water in the extracellular compartment.	ml
	61	C22_14	ICW	Intracellular water	Amount of water in the intracellular compartment.	ml
	62	C22_14	Hidr	Hydration	Dynamic equilibrium between intake and discharge of human water.	ρ
	63	C22_14	R	Resistance	Body opposition to current flow, related to tissue hydration.	
	64	C22_14	Z	Impedance	Body impedance, is related to total body water and cellular mass.	
	65	C22_14	PA	Phase angle	Direct measurement of cell integrity and the distribution of water within and outside the cell membrane.	°
Blood chemistry	66	C22_14	Non_HDL	Non HDL cholesterol	Total cholesterol amount that is not HDL.	mg/dL
	67	C22_14	Phos	Phospholipids	Serum phospholipids reflect the cellular membrane composition.	mg/dL
	68	C22_14	apoB	apolipoprotein B	Major lipid transport protein in VLDL, IDL and LDL.	mg/dL
	69	C22_14	apoA	apolipoprotein A	Major lipid transport protein in HDL.	mg/dL
Spirometry	70	C22_14	NO	Nitric oxide exhaled fraction	Biomarker for the diagnosis, follow-up and as a guide to therapy in patients with asthma.	ppb
	71	C22_14	CO	Carbon monoxide exhaled fraction	Biomarker of pathophysiological states, including smoking status, and inflammatory diseases of the lungs.	ppm
	72	C22_14	COHb	Carboxyhemoglobin	Hemoglobin irreversibly bound to CO	%
	73	C22_14	FVC	Forced vital capacity	Maximum amount of air expelled from the lungs after a maximum inhalation.	L
	74	C22_14	FEV1	Forced expiratory volume 1st second	Volume of air expelled in the first second after a forced inhalation.	L
	75	C22_14	Coadj	Carbon monoxide diffusion altitude adjusted	Ability of the lungs to transfer gas from inhaled air to the red blood cells in pulmonary capillaries.	ml/min/mmHg
	76	C22_14	AV	Alveolar volume	Alveolar volume.	L
	77	C22_14	kCO	Carbon monoxide diffusion constant	Index of the efficiency of alveolar transfer of carbon monoxide.	DLco/V
	78	C22_14	TLC	Total lung capacity	Volume of air within the lungs.	L
	79	C22_14	RV	Residual volume	Volume of air remaining after forceful expiration.	L
Molecular biology	80	C22_14	C-pep	C peptide	Inert peptide produced in endogenous insulin maturation, equimolar to insulin.	pg/ml
	81	C22_14	Ghre	Ghrelin	Incretin.	pg/ml
	82	C22_14	GIP	GIP	Incretin.	pg/ml
	83	C22_14	GLP1	GLP-1	Incretin.	pg/ml
	84	C22_14	Glcgn	Glucagon	Hyperglycemic hormone produced by alpha cells.	pg/ml
	85	C22_14	Lept	Leptin	Adipokine.	pg/ml
	86	C22_14	PAI1	Plasminogen activator inhibitor-1	Endothelial anti-fibrinolytic protein.	pg/ml
	87	C22_14	Resistin	Resistin	Adipokine.	pg/ml
	88	C22_14	Visfatin	Visfatin	Adipokine.	pg/ml
	89	C22_14	GST	Glutation S transferase	RedOx enzyme plasmatic activity.	nmol/mg prot/min
	90	C22_14	Argi	Arginase	Activity of arginase, indirect regulator of NO production.	mmol/mg prot/min
	91	C22_14	MPO	Myeloperoxidase	Neutrophil enzyme in the phagosome.	U/ml
	92	C22_14	MDA	Malondialdehyde	Oxidative stress end product.	μm
	93	C22_14	ICAM	ICAM1	Endothelial activation biomarker.	ng/ml
	94	C22_14	VCAM	VCAM-1	Endothelial activation biomarker.	ng/ml
	95	C22_14	Endo	ENDOTHELIN-1	Endothelial activation biomarker.	pg/ml
	96	C22_14	oxLDL	Oxidized LDL	LDL with changes attributable to oxidative stress.	ng/ml
	97	C22_14	PON1	Paraoxonase 1	Hydrolytic enzyme that metabolizes organophosphates and protects LDL against oxidation.	nmol p-nitrophenol/mg prot/min
	98	C22_14	CC16	cc16	Clara cell protein 16.	ng/ml
	99	C22_14	Car-G	Carbonyl groups	Biomarker of late damage to protein, 60% attributable to MDA reaction.	nmol osazonas/mg prot
	100	C22_14	LHOO	Lipoperoxide	Oxidative stress lipid damage biomarker	nMol/ml

#### C22_14 Database

##### Ethical and human research considerations

This study was carried out in accordance with current regulation of the Mexican Official Normativity, NOM-012-SSA3-2012. The Ethics Committee of the “Instituto Nacional de Enfermedades Respiratorias” (INER) approved the procedures and protocols for this study as project **C22_14**, all the participants provided a written informed consent.

##### Demographic description of the participants

134 participants from Mexico City and surroundings were evaluated, corresponding to 43 men and 91 women with an age ranging from 25 to 67 years old (median age = 46 years old). Overweight and obesity were highly prevalent, being present in 42% and 39% of the participants, respectively. 81 independent variables were measured through anthropometry, bioimpedance, spirometry, complete blood count, blood chemistry and ELISA (see list of variables in Table 1). Several derived variables of common use in medical practice were calculated for the database (see list of derived variables in Table 2).

**TABLE 2:** Description of derived variables.

**ID**	**Name**	**Variable**	**Description**	**Units**	**Formula**	**References**
101	MAP	Mean arterial pressure	Area under the pressure/time curve, divided by the cardiac cycle time.	mmHg	$\frac{\mathrm{SBP}+2\,\mathrm{DBP}}{3}$	DeMers and Wachs, 2020
102	BMI	Body mass index	Anthropometric index of adiposity.	kg/m^2^	$\frac{\mathrm{Wt}}{{\left(\mathrm{Ht}\right)}^{2}}$	Jabłonowska-Lietz et al., 2017
103	BFp	Body fat percentage	Proportion of fat to the weight.	%	$\frac{\mathrm{BF}}{\mathrm{Wt}}*100$	Freedman et al., 2012
104	FFMp	Lean mass proportion	Proportion of lean mass to the weight.	%	$\frac{\mathrm{FFMp}}{\mathrm{Wt}}*100$	Welch et al., 2016
105	BFI	Body fat index	Proportion of fat to the height.	%	$\frac{\mathrm{Hip}}{{\left(\mathrm{Ht}\right)}^{3}}-18$	Jabłonowska-Lietz et al., 2017
106	LMI	Lean mass index	Proportion of lean mass to height.	kg/m^2^	$\left(\frac{\mathrm{FFM}}{{\mathrm{Ht}}^{3}}\right)$	Welch et al., 2016
107	SMMp	Skeletal muscle mass proportion	Proportion of muscle to the weight.	%	$\frac{\mathrm{SMM}}{\mathrm{Wt}}*100$	Fukuoka et al., 2019
108	TBWp	Total body water proportion	Estimated percentage water in the body to the weight.	%	$\frac{\mathrm{TBW}}{\mathrm{Wt}}*100$	Chumlea et al., 2001
109	ECWp	Extracellular water proportion	Percentage of water in the extracellular compartment to the total body water.	%	$\frac{\mathrm{ECW}}{\mathrm{TBW}}*100$	Silva et al., 2008
110	ICWp	Intracellular water proportion	Percentage of water in the intracellular compartment to the total body water.	%	$\frac{\mathrm{ICW}}{\mathrm{TBW}}*100$	Silva et al., 2008
111	Neup	Neutrophils proportion	Percentage of innate immunity white blood cell respect to Leuk.	%	$\frac{\mathrm{Neu}}{\mathrm{Leuk}}*100$	Riley and Rupert, 2015
112	Lymp	Lymphocytes proportion	Percentage of adaptative immunity white blood cell respect to Leuk.	%	$\frac{\mathrm{Lympho}}{\mathrm{Leuk}}*100$	Riley and Rupert, 2015
113	Monop	Monocytes proportion	Percentage of innate immunity macrophage precursor to Leuk.	%	$\frac{\mathrm{Mono}}{\mathrm{Leuk}}*100$	Riley and Rupert, 2015
114	Eosp	Eosinophils proportion	Percentage of allergic and parasitic response blood cell to Leuk.	%	$\frac{\mathrm{Eos}}{\mathrm{Leuk}}*100$	Riley and Rupert, 2015
115	Basop	Basophiles	Percentage of least common type of granulocyte respect to Leuk.	%	$\frac{\mathrm{Baso}}{\mathrm{Leuk}}*100$	Riley and Rupert, 2015
116	Cast1	castelli1	An index of cardiovascular risk based on cholesterol.	$\frac{\mathrm{mg}/\mathrm{dL}}{\mathrm{mg}/\mathrm{dL}}$	$\frac{\mathrm{Col}}{\mathrm{HDL}}$	Vargas et al., 2014
117	Cast2	castelli2	An index of cardiovascular risk based on LDL.	$\frac{\mathrm{mg}/\mathrm{dL}}{\mathrm{mg}/\mathrm{dL}}$	$\frac{\mathrm{LDL}}{\mathrm{HDL}}$	Vargas et al., 2014
118	HOMA-IR	Homeostatic model assessment	Fasting insulin resistance index.	mmol/L *mIU/L	$\frac{\mathrm{Gluc}*\mathrm{Ins}}{22.5}$	Matthews et al., 1985
119	HOMA-β	Homeostatic model assessment	Evaluate the functioning capacity of the beta cells of pancreas	$\frac{\mathrm{mIU}/L}{\mathrm{mmol}/L}$	$\frac{20*\mathrm{Ins}}{\mathrm{Gluc}-3.5}$	Matthews et al., 1985
120	BUN	Blood ureic nitrogen	Equivalent to urea.	mg/dL	$\frac{\mathrm{Urea}}{2.14}$	Arihan et al., 2018
121	FVCp	Predicted vaule of forced vital capacity	Total amount of air exhaled. Evaluates Lung function during spirometry.	%	*TCPI*+*RVE*+*RVE*	Physiopedia, 2020
122	FEV1p	Predicted vaule of forced expiratory volume 1^st^ second	Volume of air expelled in the first second after a forced inhalation.	%	0.84*FVC−0.23	Gólczewski et al., 2012
123	rel	FEV1/FVC ratio	Is the amount of air exhaled in the first second divided by all of the air exhaled during a maximal exhalation. It is considered a bronchial obstruction marker.	%	$\frac{\mathrm{FEV1}}{\mathrm{FVC}}$	Gólczewski et al., 2012
124	COdlp	Carbon monoxide diffusion altitude adjusted	Measure of the conductance of gas transfer from inspired gas to the red blood cells.	mmol/ (min kPa)	COdl measured * (1.0 + 0.0035 (PaO2 - 120))	McCormack, 2020
125	ALVp	Predicted value of alveolar volume	Alveolar volume.	%	$\begin{array}{c} \left[\frac{F\,I\,C\,H\,4}{F\,A\,C\,H\,4}\right]*\left[\begin{array}{c} V\,i\,n\,s\,p-\left(\begin{array}{c} V\,d\,i\,n\,s+V\,D\,a\,n\,a\,t \end{array}\right) \end{array}\right] \end{array}$	Prediletto et al., 2007
126	kCOp	Predicted value of carbon monoxide diffusion constant	Index of the efficiency of alveolar transfer of carbon monoxide.	%	$\frac{\mathrm{DA}}{\mathrm{LV}}$	Vermesi et al., 2018
127	TCPp	Predicted value of total lung capacity	Proportion of predicted volume of air within the lungs.	%	Functional Residual Capacity + Inspiratory Capacity	Vaz Fragoso et al., 2017
128	RVp	Predicted value of residual volume	Proportion of predicted volume of air remaining after forceful expiration.	%	TCP – Inspiratory vital capacity	Vaz Fragoso et al., 2017

##### Measurement of physiological variables

Approximately 10 mL of venous blood was taken from each participant in fasting conditions and stored in darkness throughout the biospecimen handling process. EDTA or heparin was used as an anticoagulant according to the determinations to be made. The samples were centrifuged, plasma was obtained and routine clinical analysis was performed to know the health state of individual subjects. The set of bioclinical tests included hematologic analyses, biochemistry, C-reactive protein, which were carried out in the local clinical laboratory in compliance with current quality standards. Additionally, analyses by ELISA were performed. Spirometry was carried out in the Clínica de Ayuda para dejar de Fumar at the INER.

#### Project_42 Database

##### Ethical and human research considerations

This study was carried out in accordance with current regulation contained in the Mexican Official Normativity, NOM-012-SSA3-2012. The Ethics Committee of the Facultad de Medicina of the Universidad Nacional Autónoma de México (UNAM) approved the procedures and protocols for this study as project FM/DI/023/2014. All the participants provided a written informed consent.

##### Demographic description of the participants

This sample was based on first and second year students at the School of Medicine at the UNAM, all living in Mexico City and its surroundings. 69% of the sample were women, with an age ranging from 18 to 28 years old (mean age of 20 ± 2 years-old). 54 independent variables were measured through anthropometry, bioimpedance, hematic biometry, blood chemistry (see list of variables in Table 1). Derived variables used commonly to characterize meaningful relations between variables are present in the database (see list of derived variables in Table 2).

##### Measurement of physiological variables

After a medical check-up, all samples and anthropometric measurements were realized in fasting conditions from 7:00 to 9:00 h. Anthropometric measurements were performed employing the corresponding WHO guidelines. All participants were advised to abstain from alcohol and other substances 24 h prior to the measurements. All blood samples were stored at 4°C and processed the same day. A full description of the methods employed for this dataset is available in Barajas-Martínez et al. (2020).

### Data Processing

Databases were constructed manually in excel and validated at random as quality control.

All the physiological variables obeyed asymmetric and leptokurtic distributions, such that the median value (Me) was considered to be the best measure of the distribution center, and the range (difference between maximum (*Max*) and (*Min*)) to be the best representation of the dispersion. For each variable from the data we obtained the normalized value *x*_*i*_ applying the following normalization to the original data *V*_*i*_ :

$$x_{i}=\left(\frac{V_{i}-M\,e}{M\,a\,x-M\,i\,n}\right)$$

Outliers and implausible data were screened using the ROUT method where *Q* = 1%. Given the leptokurtic distributions, both databases presented various outlying values. However, most of these outliers were within expected ranges of biological variability. In the **C22_14** database no outliers were discarded. In the **Project_42** database, 3 values each were discarded for waist circumference, systolic blood pressure, and glucose, 2 values for hip and 1 value each for creatinine, arm and wrist temperature.

### Network Construction

**FIGURE 1:**
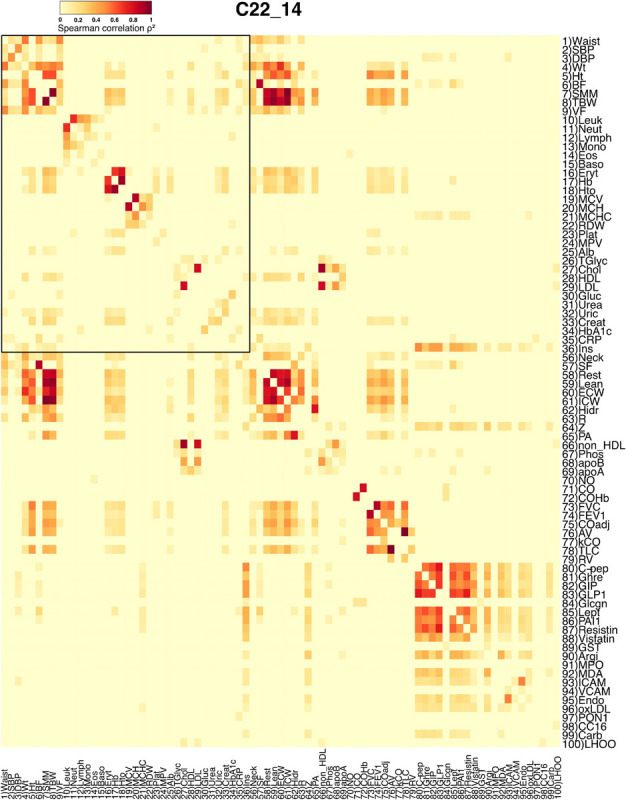
Adjacency matrix for the C22_14 database. Spearman correlation values were squared to obtain only positive values. The strength of each link is shown in the heatmap as a heat gradient. Numerical ID and short name are presented next to rows and below columns. The shared physiological variables between both databases are encased within the black rectangle in the upper left side of the heatmap.

**FIGURE 2:**
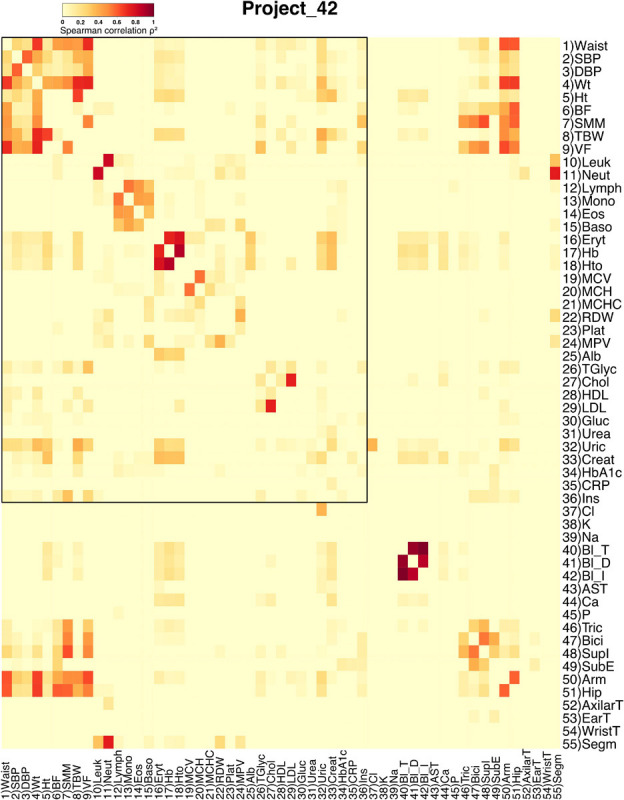
Adjacency matrix for the Project_42 database. Spearman correlation values were squared to obtain only positive values. The strength of each link is shown in the heatmap as a heat gradient. Numerical ID and short name are presented next to rows and below columns. The shared physiological variables between both databases are encased within the black rectangle in the upper left side of the heatmap.

All physiological variables were tested for normality using the Shapiro-Wilk test and they were screened as well for extreme values. Since the data sets were not normally distributed and presented outlying values within ranges of biological variability, the Spearman rank correlation ρ (Batushansky et al., 2016) was selected as a measure of correlation (Figures 1, 2). The Spearman rank correlation is a nonparametric measure of the statistical dependence between the rank values of the variables considering monotonic relationship (not necessarily linear) and is not affected by the normalization. For each pair of physiological variables, *X* and *Y*, rank (*rk*_*X*_, *rk*_*Y*_, respectively) and standard deviation (σ_*rk_X*_, σ_*rk_Y*_) were evaluated, and the Spearman rank correlation was calculated as the ratio between covariance (*co*ν) and deviations:

$$\rho =\frac{c\,o\,\nu \,\left(r\,k_{X},r\,k_{Y}\right)}{\sigma_{r\,k_{X}}\,\sigma_{r\,k_{Y}}}$$

**FIGURE 3:**
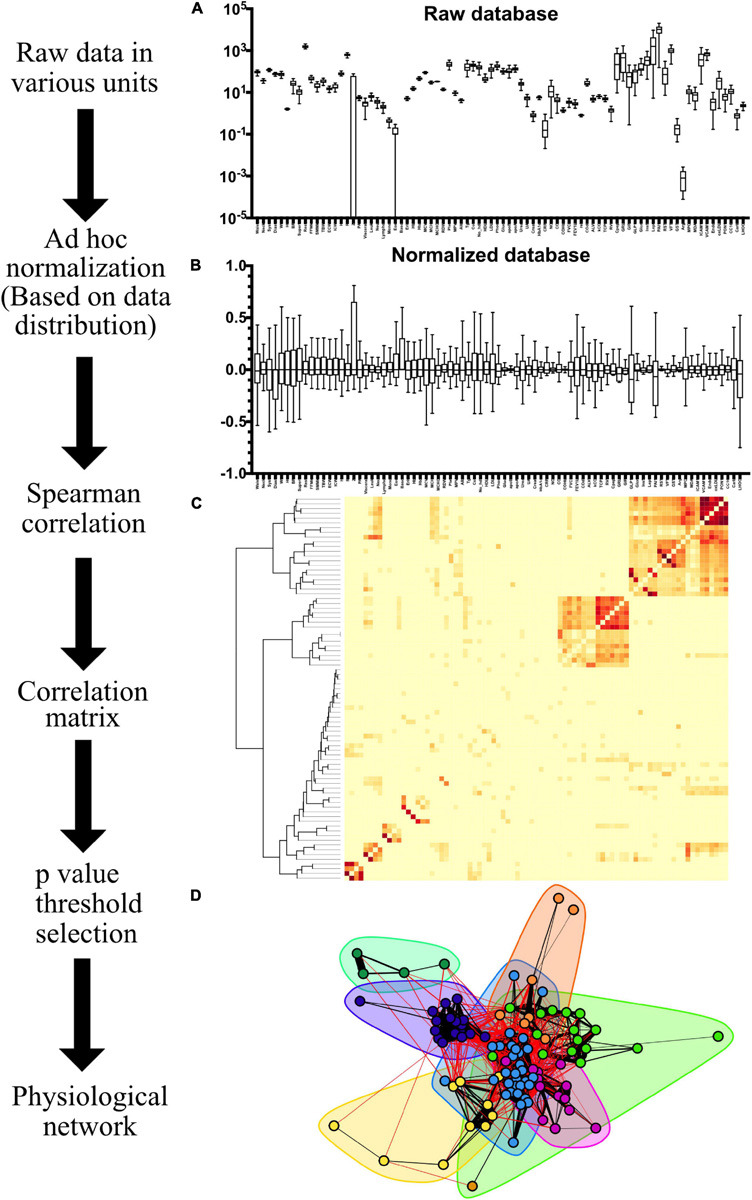
Network construction workflow. The range and distribution of values recorded in the raw database as Tukey box-plots are shown in **(A)**. Variables are normalized, resulting in the box-plots shown in **(B)**. The resulting Spearman ρ correlation matrix, with squared values to represent correlation between variables regardless of sign, is shown as an adjacency matrix heatmap in **(C)** with a hierarchical dendrogram on the left. After choosing a *p*-value threshold to discard insignificant links, a network can be constructed as illustrated in **(D)**. Network structures can be enriched with several features, e.g., clusters (shadowed areas) and strength of the spearman correlation (width of the links).

To test if the Spearman rank correlation is significantly different from zero, a Student’s *t*-distribution with (*n*-2) degrees of freedom was employed. Significant correlations were established below a threshold value of *p* < 0.001, indicating that the relation does not support the null hypothesis that the independent and dependent variables are unrelated. The Spearman rank correlation coefficient ρ was squared in order to obtain only positive values (Figure 3). An adjacency matrix was constructed with matrix elements corresponding the ρ coefficients between each pair of physiological components such that the resulting network was weighted (Figure 3). Data-set normality testing, linear regression and chi-squared tests for trends were realized with Prism 8.1.2(277), GraphPad Software, La Jolla, CA, United States, www.graphpad.com. For the network construction RStudio, an R language programming suite and igraph package were employed (Csárdi et al., 2016; R Core Team, 2020; RStudio Team, 2020). A glossary of terms used in this paper is given in Table 3.

**TABLE 3:** Glossary. A brief description for quick reference of network’s specialized terms.

**Glossary**	**Symbol**	**Definition**	**R_package::function**	**References**
Graph	G = (V, E)	A network, composed of a set of nodes (V) and links (E).		
Adjacency matrix	A	An array of rows and columns that contains the connections of the network		
Subgraph	S ⊆ V	A subset of nodes and their links contained in the original network	igraph::induced.subgraph	
Vertex	V	A node		
Edge	E	A link		
Centrality	C	A measure that describes a node’s overall role in the network		Borgatti and Everett, 2006.
Degree		Number of links that a node has	sna::degree	Freeman, 1978.
Strength		The sum of the weights of the links attached to a node	igraph::strength	Barrat et al. (2004).
Flow	F	A measure that describes the strength of the links in a path between nodes		
Radial measures	Those centralities that are based on pair-wise connections			Borgatti and Everett, 2006.
Eigencentrality	v = λ−1Av	This centrality of each node is proportional to the sum of the centralities of those nodes to which it is connected.	sna::evcent	Katz (1953).
Hub score	Eigen-centrality from A*t(A)	Eigencentrality of the matrix that takes into account only out-going links	igraph::hub_score	Kleinberg and Tardos (1998)
Medial measures	Those centralities that are based on the number of walks that pass through a node			Borgatti and Everett, 2006.
Flow betweenness		The amount of flow mediated by a given node. This illustrates the **gate-keeping** role of a node i.e. the potential to disconnect the network.	sna::flowbet	Koschützki et al. (2005).
Cluster, community	A set of nodes with many links between themselves and few nodes to the outside of the community (the rest of the network).			Blondel et al., 2008.
Clique	A subgraph where all nodes are fully connected between themselves.			Eppstein and Strash, 2011
Largest clique		The clique(s) with the largest size possible contained in the network	igraph::largest.cliques	Eppstein and Strash, 2011
Louvain, cluster		An algorithm for finding communities that works through modularity optimization.	igraph::cluster_louvain	Blondel et al., 2008.
Spinglass, cluster		An algorithm for finding communities based on simulated annealing and a spin-glass model.	igraph::spinglass. community	Reichardt and Bornholdt (2006)
Topology	The structural characteristics of the network			
Size		The number of nodes in the network		
Density		The ratio of links that are present in a network to all the possible edges it could contain.	igraph::graph.density	Wasserman and Faust (1994).
Reciprocity		The ratio of bidirectional links in a directed graph.	igraph::reciprocity	
Characteristic path length	L	The average of all the shortest paths between each pair of nodes in the network	igraph::average.path.length	West (1996).
Transitivity, local		Transitivity and clustering coefficient are two slightly different ways of counting triangles in a network. Both can be local, when only one node and their neighbors are considered, or global, when the whole network is considered. It represents the ratio of all the triangles present to all the possible triangles in the network.	igraph::transitivity	Barrat et al. (2004)
Transitivity, global	T		igraph::transitivity	Barrat et al. (2004)
Clustering coefficient, local			DirectedClustering::ClustF	Fagiolo (2007)Onnela et al. (2005)
Clustering coefficient, global	CC		DirectedClustering::ClustF	Fagiolo (2007)Onnela et al. (2005)
Small world index	SWI	A measure that describes the relation between CC and L in a network against what would be expected in a random network.	qgraph::smallworldIndex	Watts and Strogatz (1998).
Scale-free fitting index	SFFI R^2^	A measure of how well a network complies with an scale-free fitting linear regression.	WGCNA:: scaleFreeFitIndex	Langfelder and Horvath, 2008.

Nodes within a network can be ranked according to several centrality definitions that fall into two main groups, radial measures and medial measures. These centrality values allow for a direct comparison of either the influence of nodes (radial measure) or gatekeeping (medial measure) within the network (Borgatti and Everett, 2006). Eigencentrality corresponds to the value of the first eigenvector of the graph adjacency matrix and was interpreted as a measure of influence within the undirected networks. Inferring causality exclusively from centrality within networks requires caution, although eigencentrality has been found to be the best centrality measurement for this purpose, especially for small networks with less than 30 nodes (Dablander and Hinne, 2019). Furthermore, eigencentrality is resilient to incomplete sampling of the underlying network (Costenbader and Valente, 2003). For radial measures eigenvectors were selected for the undirected networks and hubscores for the directed networks, whereas for medial measures flow betweenness was used. Flow betweenness was used as a measurement of intermediation within the network. These values were obtained using the SNA package (Butts, 2019). Univariate conditionally uniform graph tests (CUG test), more in particular the cug.test function from the SNA package, were employed in order to test whether the eigencentrality and flow betweenness values obtained would be seen in a random graph with the same number of vertices, edges or dyads. Assortativity of these centralities, i.e., the tendency of nodes with similar centrality to link together, was calculated. NetSwan package was used for studying network robustness, resilience, and vulnerability. Differences were assessed with a paired Friedman’s test using Dunn’s *post hoc* test. Topological properties were assessed as follows: density, reciprocity and characteristic path length of the networks were calculated using the igraph package. For the calculation of the weighted transitivity and the clustering coefficient in directed and undirected weighted networks the DirectedClustering package was employed (Clemente and Grassi, 2018). CUG tests were also performed for network density, efficiency, transitivity and characteristic path length. The small world index and smallworldness as calculated by qgraph, were used as a summary metric of the network topology (Watts and Strogatz, 1998). Scale-free fitting index was calculated to show fit to scale-invariant distribution using WGCNA package (Langfelder and Horvath, 2008).

In order to generate a common layout to both networks the edge lists of both networks were merged. The resulting network contained all 100 nodes from both datasets with their corresponding edges. This network was outlined with Fruchterman-Reingold force-directed layout (Fruchterman and Reingold, 1991). As a result of this procedure, the relative position of each of the shared nodes between the different networks was the same. This allowed an easy side-by-side contrast between networks. When clusters were collapsed into nodes, they were placed in the location of the node with greatest strength in each cluster to retain the general arrangement of the network.

#### Cluster Detection

Determining whether a natural division of nodes is present in a network entails practical and useful insight of the studied system that is not accessible in reductionist approaches. A cluster is a set of nodes with many edges inside and few edges outside the cluster. This condition must also meet the requisite of surpassing what would be expected in an equivalent network where links are placed at random (Newman, 2006). This is tested through positive values of modularity in a network. Clusters can be detected by using a suitable algorithm, that groups vertices within a graph that are more densely connected to one another than to other vertices (Figure 3; Csárdi et al., 2016). There are several alternative algorithms for discovering communities of vertices within graphs. In the present contribution, 2 clustering algorithms were employed that are included in the igraph package, Louvain and MAP (Blondel et al., 2008; Rosvall and Bergstrom, 2008). The results were compared using the igraph::compare function for the calculation of the Rand Index (Rand, 1971) and variation of information (Meilă, 2007). The results of this unsupervised clustering were then examined against current literature to find the functional systems that best described the nodes.

##### Construction of clusters based on unsupervised classifiers

Communities may also be found through walks, simulated annealing, or greedy algorithms, that are supposed to converge iteratively to the best result. 2 clustering algorithms were employed that are included in the igraph package, Louvain, a greedy algorithm, and MAP, a method based on walks and information theory (Blondel et al., 2008; Rosvall and Bergstrom, 2008). The Louvain algorithm optimizes modularity, the ratio between density of links inside the community, compared to the links between communities. To do so, at first, each node is a community of its own. With each step, nodes are re-assigned to communities in a local greedy way. Each node is placed in the community where modularity is increased most. When all nodes are assigned, each community follows the same merging and relocating procedure until modularity cannot be further optimized (Blondel et al., 2008). In contrast, InfoMap clustering tries to minimize the description length of a random walker’s movements on a network (Rosvall and Bergstrom, 2008). To increase the detail of the generated clusters, each cluster subgraph was clustered as an independent network, generating subclusters. Additionally, force-directed layouts such as a Linear logarithmic layout (Linlog) and a Fruchterman-Reingold layout may complement the representation of the community structure of a network (Noack, 2009).

## Results

### Network Topology Changes With Significance Threshold

**FIGURE 4:**
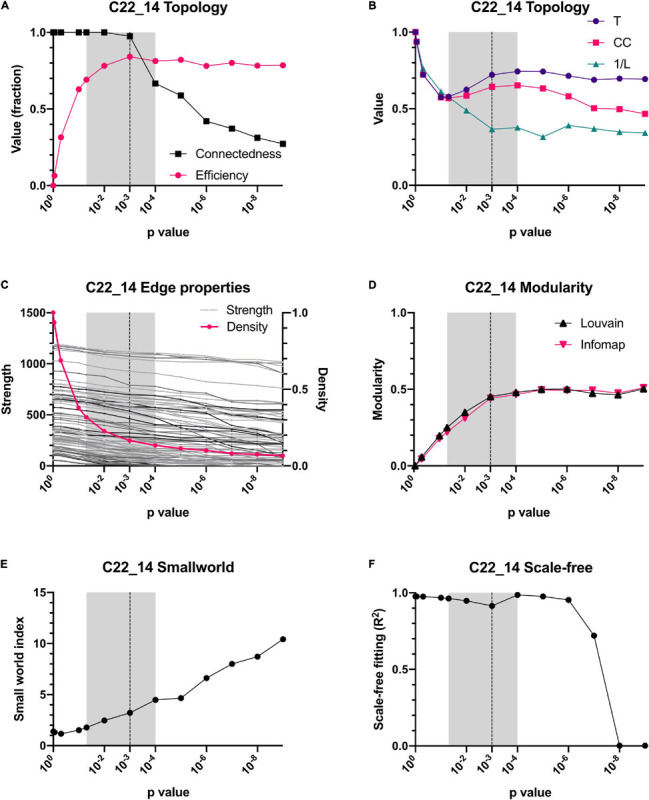
Dependence of topology on the *p*-value threshold for the C22_14 database. For all panels, shadowed areas show the windows employed for examining the networks, from *p* < 0.05 to *p* < 0.0001. Our selected threshold, *p* < 0.001, is shown as a vertical dotted line. Relationship of connectedness and efficiency of the network is shown in **(A)**. Topology indicators such as characteristic path length (L), global Barrat’s weighted transitivity (T) and clustering coefficient (CC) are shown in **(B)**. A comparison between density (number of connections) and strength (sum of weights of links for each node) is presented in **(C)**. Modularity quantified by 2 different clustering strategies, Louvain clustering and InfoMAP, increases as a function of the *p*-value threshold **(D)**. Small-world index increase with *p*-value threshold **(E)**. Node’s degree frequency distribution fitting to a scale-free model is shown in **(F)**.

**FIGURE 5:**
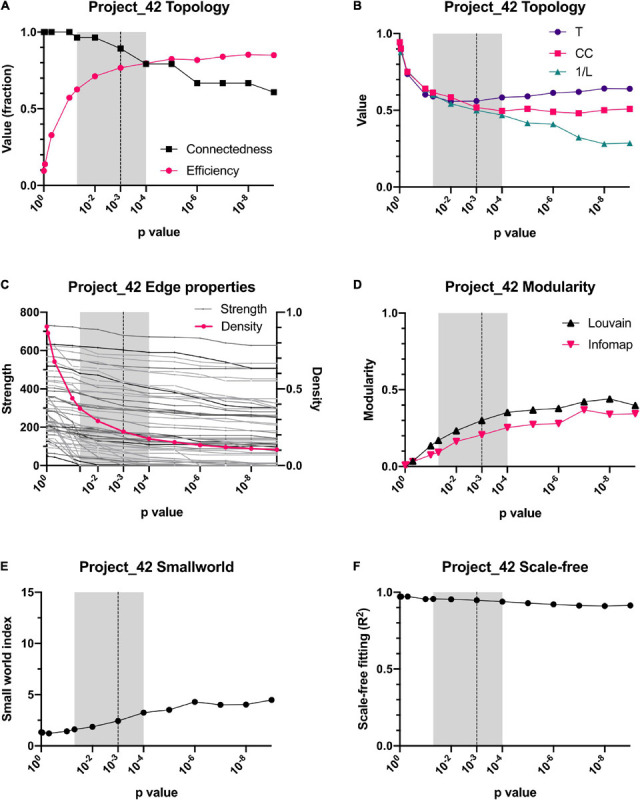
Dependence of topology on the *p*-value threshold for the Project 42 database. For all panels, shadowed area shows the window we employed for examining networks, from *p* < 0.05 to *p* < 0.0001. Our selected threshold, *p* < 0.001, is shown as a vertical dotted line. Relationship of connectedness and efficiency of the network is shown in **(A)**. Topology indicators such as characteristic path length (L), global Barrat’s weighted transitivity (T) and clustering coefficient (CC) are shown in **(B)**. The decrease of networks number of connections (density) is contrasted against the sum of the links weight for each node (strength) is presented in **(C)**. Modularity increase with *p*-value threshold is presented in through 2 clustering strategies, Louvain clustering and InfoMAP **(D)**. Small-world index increase with *p*-value threshold is presented in **(E)**. Node’s degree frequency distribution fitting to a scale-free model is shown in **(F)**.

The reliability of the present approach was tested by checking whether data normalization or a variation in the *p*-value threshold resulted in substantial changes in the network topology or in the centrality of individual variables (Figures 4, 5). Spearman correlation matrices resulted to be very similar and largely independent form of data treatment. This is an indication of the robustness of the correlations between variables. As intuitively expected, without a threshold, the result is a fully connected network. However, by lowering the *p*-value required to indicate a significative relationship between two variables, the topology of the network changed abruptly, until reaching a value *p* < 0.05 (Figures 4A,C, 5A,C). For constructing physiological networks, connectivity is a desirable feature, since little can be said of isolated nodes. On the other hand, also a deletion of redundant links is needed because correlations arise from collinearity of the variables. The best compromise between these needs was *p* < 0.001. This was remarkable because the density of the network continued to decrease exponentially whereas the strength of the nodes did not decrease at the same rate. This indicated that lowering the *p*-value of the network removed preferentially the weakest links. As a result, the efficiency of the network increased, and the connectedness only decreased slightly until *p* < 0.001, where connectedness and efficiency began to decrease (Figures 4A, 5A). Characteristic path length (L) increased with the *p*-value threshold (Figures 4B, 5B). At *p* < 0.001 some relatively stable value of transitivity and clustering coefficient was obtained (Figures 4B, 5B). Moreover, although transitivity and characteristic path length remained similar, small world index increased steadily (Figures 4E, 5E). This indicated that the underlying topology of the network is not a product of the threshold but is actually a phenomenon of high significance. The *R*^2^ value for fitting a scale-free network was above 90% until *p* < 10^–6^, when it decreased abruptly (Figure 4F). Regarding centrality measurements, eigenvectors were stable across all the range of *p*-values, making it a centrality measure robust to any data processing. On the other hand, betweenness centralities were dependent on walks, paths or flows that, being macro-scale properties, relied on the overall structure of the network. As such, flow betweenness was more variable. The best Freeman centralization for the flow betweenness was also reached at *p* < 0.001. Despite the similarity of the correlation matrices of raw and normalized data, modularity was visibly improved by normalization procedures. This indicated an increase in intra-cluster correlations and decrease of inter-cluster correlations. It was concluded that data normalization provided the best results to find community structures in these networks.

### Network Comparison

At the selected significance threshold of *p* < 0.001, **C22_14** database resulted in a correlation matrix with 523 links, while **Project_42** had 368 links. Overall, links in **C22_14** database were stronger, but this difference was small (mean difference 8 ± 1.4, *p* < 0.0001). In contrast, node strength (weighted degree, the sum of links weight) was greater in **Project_42** (mean difference 131 ± 46, *p* < 0.001). However, strength of both nodes and edges were highly correlated in the 36 nodes and 80 links that both networks had in common (Spearman’s rho = 0.69, *p* < 0.0001 for nodes and Spearman’s rho = 0.5, *p* < 0.0001 for edges).

**FIGURE 6:**
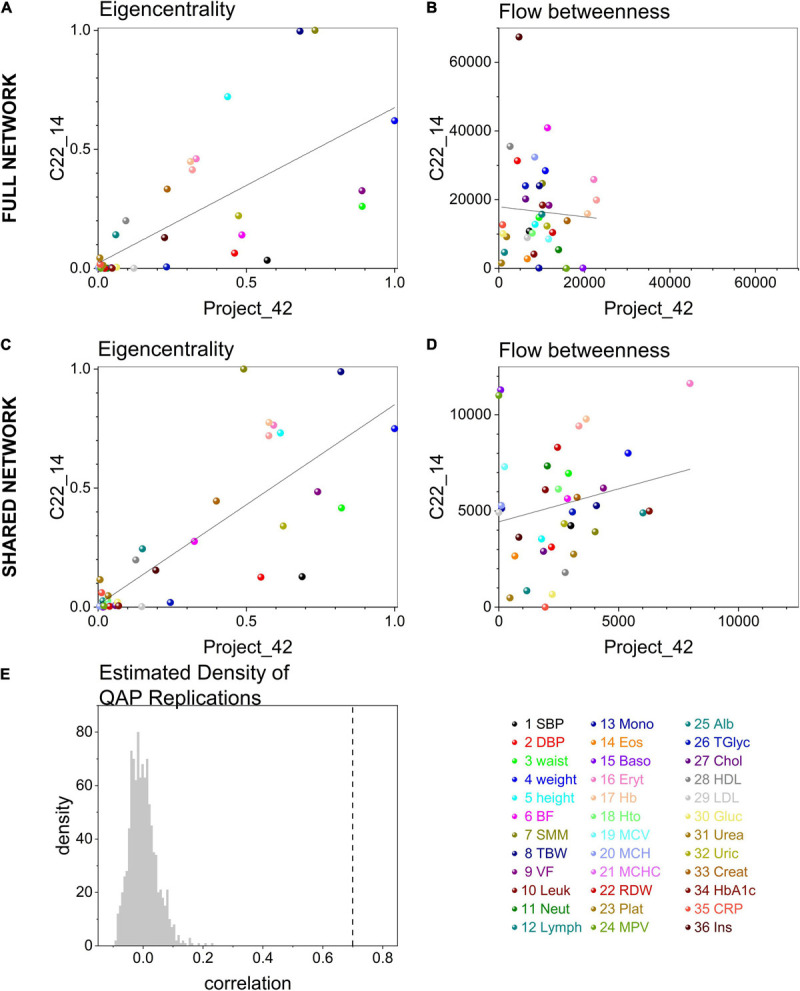
Matching between centrality measures in networks. Centrality measures, eigencentrality and flow betweenness, correlation between **C22_14** and **Project 42** full networks are shown in **(A)** and **(B)** respectively. After extracting the subgraph of the matching 36 shared physiological variables in both datasets the comparison was repeated in **(C)** and **(D)**. Linear regression (continuous curves) with 95% confidence intervals (dashed curves) are shown together with the values of all physiological variables (dots). The color of the dot indicates the specific variable. The density of the distribution of Montecarlo draws for correlation between networks in Quadratic Assignment Procedure test (QAP test) is presented in **(E)**. The dashed line indicates the correlation values between **C22_14** and **Project_42** shared networks.

To test whether node centrality measures were similar between networks the values obtained in the full networks and in the shared network were compared, built from the common subset of variables studied in both databases. For full networks eigencentrality values were similar for both datasets (Spearman’s rho = 0.74, *p* < 0.0001) while flow betweenness was dependent on the specific network (Spearman’s rho = 0.03, *p* = 0.8, see Figures 6A,B). Similar to the comparison of the full networks, when comparing networks comprising only shared nodes large correlations were found for eigencentrality (Spearman’s rho = 0.77, *p* < 0.0001) while there was no correlation for flow betweenness (Spearman’s rho = 0.22, *p* = 0.2, see Figures 6C,D). These shared networks with the same number of nodes were directly comparable. The Quadratic Assignment Procedure (QAP) test showed a significant correlation which was not observed in networks with shuffled rows and columns (Figure 6E). Clusterings obtained with the Louvain method were compared with igraph::compare for the Rand Index (0.74) and variation of information (1.5), without differences using the Wilcoxon paired ranked test (*T* = 174, *W* = −6, *p* = 0.9414) and significant spearman correlation (*r* = 0.49, C.I. [0.18, 0.71], *p* = 0.002, pairs = 36). Therefore, both networks had consistent clusters as well as high correlation. This is in spite that the correlation between networks was only moderate (gcor = 69%). Nonetheless, this correlation was greater than expected from permuted networks by QAP test, indicating that this similitude was not a product of chance (Figure 6E). Differences between networks may be expected by the decrease in transitivity that we have observed with age and/or disease (Barajas-Martínez et al., 2020).

### Physiological Clusters

**FIGURE 7:**
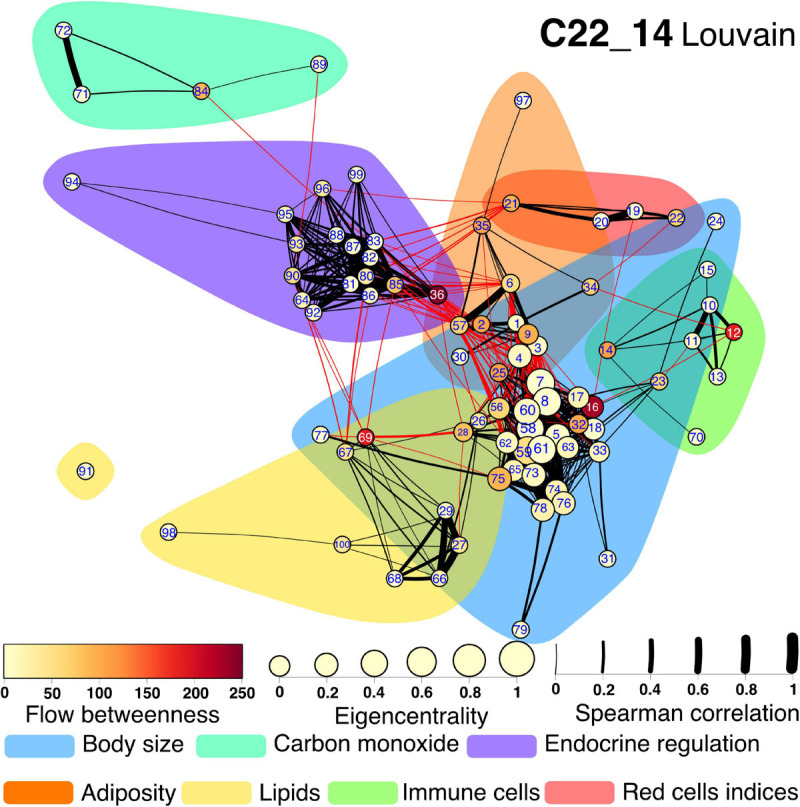
Physiological network for C22_14 database. The physiological network constructed from the correlation matrix once the *p*-value discards connections without statistical significance. Clusters are presented as shadowed areas. Links within the same cluster are black while links between clusters are red. Node centrality is represented as size for the eigencentrality and color for the flow betweenness. Edge width represents the weight of the Spearman correlation. Nodes are labeled according to Table 1 numerical ID.

**FIGURE 8:**
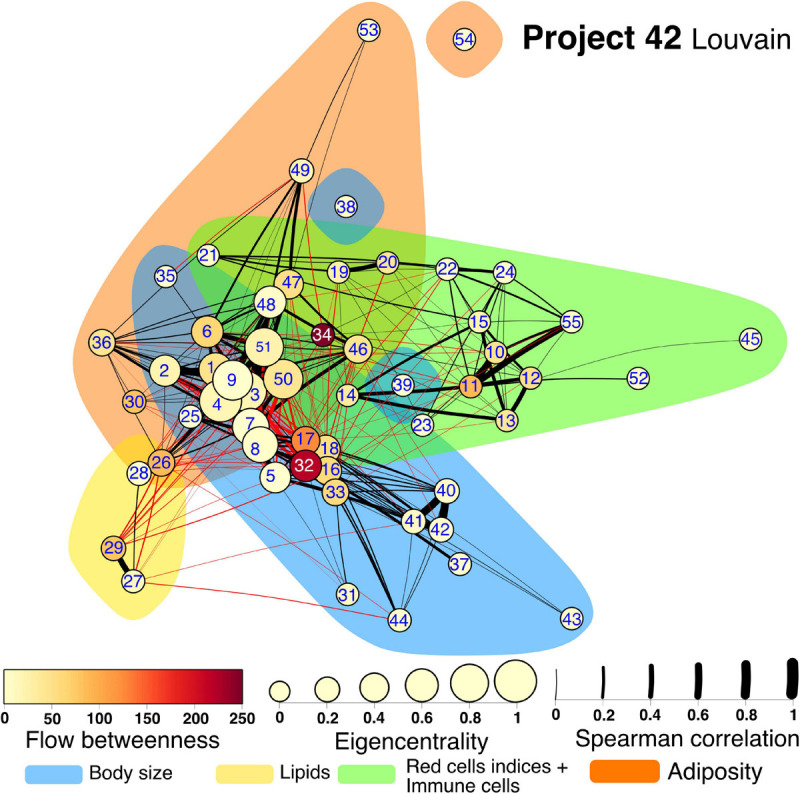
Physiological network for Project 42 database. The physiological network constructed from the correlation matrix once the *p*-value discards connections without statistical significance. Clusters are presented as shadowed areas. Links within the same cluster are black while links between clusters are red. Node centrality is represented as size for the eigencentrality and color for the flow betweenness. Edge width represents the weight of the Spearman correlation. Nodes are labeled according to Table 1 numerical ID.

Several community detection algorithms for networks were tested and evaluated through their modularity scores. Seven clusters were identified within the network (Figures 7, 8). The first cluster included anthropometric, bioimpedance and spirometry variables related with body size. This cluster has most of the nodes with high eigencentrality in the network. Most of these nodes with high influence belong to bioimpedance and spirometry variables. Three subclusters are identifiable here. First, bioimpedance and anthropometric variables, along with four biomarkers, uric acid, CRP, PON-1 and HDL. The second subcluster comprises spirometry variables, while the third includes blood biomarkers like hematocrit, erythrocytes, platelets, albumin, urea and creatinine. In contrast, only few nodes in this cluster have high flow betweenness. Platelets, erythrocytes and CRP numbers were prominent in this regard. The second cluster includes elements of endocrine regulation such as the hormones of the adipoinsular axis and endothelial activation biomarkers. Eigencentrality values in this cluster are low, with insulin as the most influential node. This cluster has two nodes with high flow betweenness, insulin and arginase activity. Only one bioimpedance parameter is included here, the impedance value (Z). A third cluster, comprising lipidic biomarkers as well as club cell protein 16 (CC16), is present and exhibits a very low eigencentrality overall. The fourth cluster includes white blood cells and the two glycemic variables, glucose and HbA1c. This cluster has many high flow betweenness nodes. Eosinophils, lymphocytes and leukocytes were important intermediaries in the network. The fifth cluster involves the four red cell indices. From these, MCHC has a high flow betweenness. A sixth cluster around carbon monoxide is present. As this node is mostly peripheral in the network, glucagon and GST had high flow betweenness by linking these variables to the main component. For the **Project_42** database network there was one main difference related to the variable set that was employed (Figure 8). The cluster encompassing the red cells indices cluster is merged with the immune cells cluster. Figures and tables for each cluster described here are provided in supplementary material (Supplementary Figures 1–11).

**FIGURE 9:**
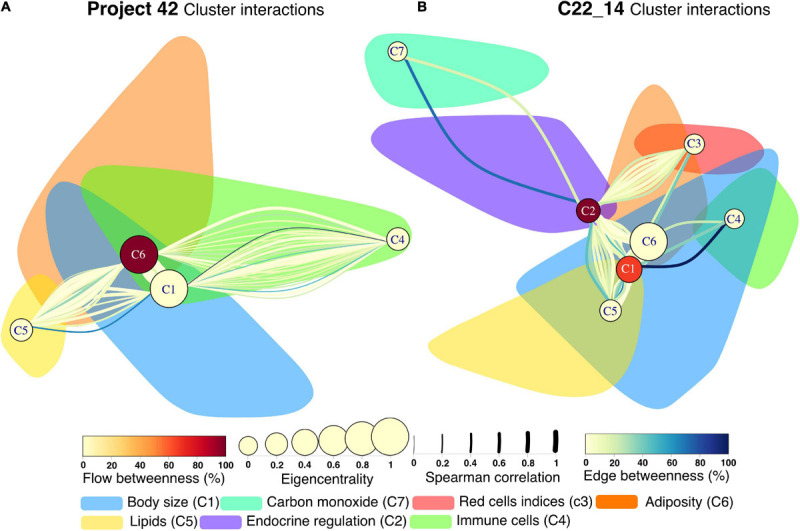
Cluster interactions network for Project 42 **(A)** and C22_14 **(B)**. Interactions between clusters are presented as a multigraph, where more than one link between two given nodes are possible. All nodes within a cluster are contracted into a single node whereas all individual links remain displayed. This new node is placed in the position of the node with greatest strength in the original network. The shadow of the original cluster remains in place for comparison with the physiological networks presented previously. The edge betweenness – the number of shortest paths that pass through a link- is presented with the color of the link, while the width represents the strength of the Spearman correlation. The new nodes centrality and flow betweenness are presented by node size and color, respectively.

Next, to highlight inter-cluster connections, that reflect the coordination between different systems within the organism, nodes inside the same cluster were contracted into a single node (Figure 9). For both networks, the body size cluster (C1) and the visceral adiposity cluster (C6) were the most closely interrelated. For the **C22_14** network, the endocrine regulation cluster (C2) is closely related to the visceral adiposity cluster (C6), the red blood cells indices (C3) and the lipids cluster (C5). For **Project_42** network, the immune cells cluster (C4) and C5 are densely connected with C1 and C6. Novel interactions between physiological systems were found. For instance, the connections between C2 and C3 in **C22_14** network represent correlations with a single red cell index, the mean corpuscular hemoglobin concentration (MCHC). Salient inter-cluster connections present in both networks were diastolic blood pressure (DBP) relation to insulin and body weight, and HbA1c correlation with total lymphocytes and red cell distribution width (RDW). Nodes that had a high number of inter-cluster connections were waist, body fat, DBP, weight, HDL and triglycerides (Figure 9). This suggests that these physiological variables are located at the crossroads between the physiological modules. This observation is reinforced by the position of waist circumference, body fat, weight, and HDL for **C22_14** and insulin for **Project_42** in the spaces between topological clusters in the Linlog Layout (Supplementary Figures 12, 13).

### Physiological network characteristics

**FIGURE 10:**
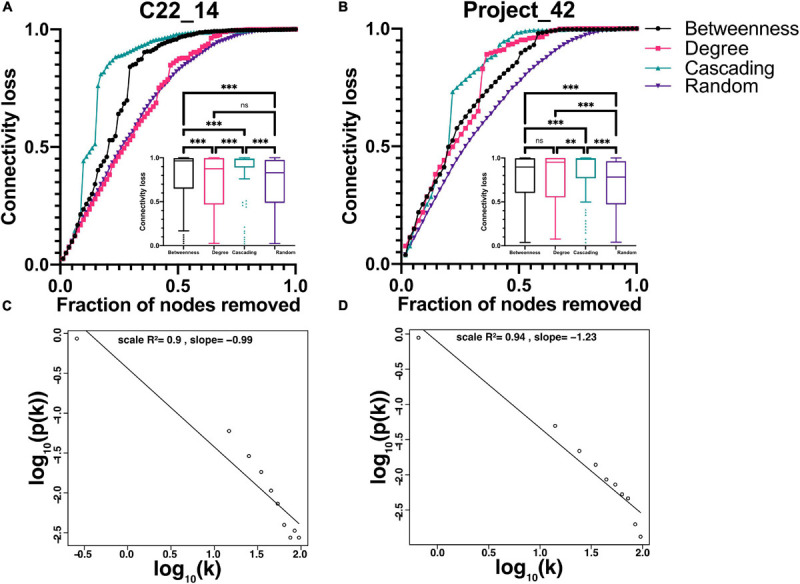
Topological characteristics of the networks. Analysis of network strengths and weaknesses is presented in **(A)** and **(B)** showing the difference between connectivity decrease in random failure (purple inverted triangles) against three different attacks, cascading (green upward triangles), betweenness (black circles) and degree (pink squares). Encased in each figure is the Tukey’s box and whiskers presentation of the data, with the Friedman’s test with Dunn’s *post hoc* test significance between groups. ** indicates *p* < 0.01, *** *p* < 0.0001. Node degree frequency distribution fitting to a scale-free model is shown in **(C)** and **(D)** for networks constructed with *p*-value thresholds of 0.001. Panel **(A)** and **(C)** for **C22_14** and **(B)** and **(D)** for **Project_42**.

The correlation matrix of the 81 unique variables studied from **C22_14** database has 523 correlations (of the possible 3240 = 80^∗^81/2) with p < 0.001, resulting in a network density of 16% (Table 4 and Figure 1). The correlation matrix of **Project_42** database has 55 unique variables studied has 368 correlations (of the possible 1485 = 54^∗^55/2) with p < 0.001 (Table 5 and Figure 2), resulting in a network density of 25% (Table 4 and Figure 1). Only myeloperoxidase (MPO) was found to be disconnected from the main component of the network for the **C22_14** network, and serum phosphorus for the **Project_42** network. With a threshold *p* < 0.01 this variable is correlated with Club cell protein 16 (CC16) and malondialdehyde (MDA). The physiological networks had an efficiency of 84% and 75%, greater than would be expected from a random network of the same size. Despite the low density of the network, it has a high transitivity of 72% and 52%, larger than would be expected in a random network with the same size, density, or number of dyads (Supplementary Figures 14, 15). Characteristic path length of 3 and 2, respectively, was higher than a random network with the same size, density, or number of dyads (Supplementary Figures 14, 15). Network architecture was evaluated for small world and scale invariance properties (Tables 4, 5). The physiological network has a small world index of 3.2, and 2 with a smallworldness of 1.9 and 1.2. Scale-free fitting index, employed as a scale invariance measurement, shows both networks approach this fitting (Tables 4, 5). As expected for a network with these topological properties, eigencentrality has a high assortativity, while flow betweenness has a low assortativity (Table 4). In turn, this assortativity, while making the network very robust against random errors, results in large susceptibility to directed attacks, particularly cascading attacks (Figures 10A,B). The elevated modularity of the physiological network results in susceptibility to betweenness-directed attacks but implies robustness to degree-directed attacks (10A and 10B). It can also be observed that the physiological network follows a scale-free distribution (Figures 10C,D). Taken altogether, the physiological network has a complex structure that satisfies the biological requirements of robustness and adaptability.

**TABLE 4:** Network topology summary for C22_14 database.

**Size**	**Edges**	**Density**	**Efficiency**	**Connectedness**
81	533	0.16	0.84	0.98

**L**	**T**	**CC**	**SWI**	**Smallworldness**

2.7	0.72	0.64	3.2	1.9

**Eigencentrality centralization**	**Eigencentrality assortativity**	**Flow betweenness centralization**	**Flow betweenness assortativity**	**Scale-free fitting index**

0.05	0.57	0.07	0.10	0.91

**TABLE 5:** Network topology summary for Project_42 database.

**Size**	**Edges**	**Density**	**Efficiency**	**Connectedness**
55	368	0.25	0.75	0.96

**L**	**T**	**CC**	**SWI**	**Smallworldness**

1.9	0.52	0.53	2.0	1.3

**Eigencentrality centralization**	**Eigencentrality assortativity**	**Flow betweenness centralization**	**Flow betweenness assortativity**	**Scale-free fitting index**

0.09	0.23	0.06	−0.10	0.98

## Discussion

Physiological networks are an area of increasing interest for the study of biological systems. These networks relate inferred interactions between systems that may be constructed from co-occurrence of observations. This co-occurrence may be observed in time, as in networks constructed from time series for dynamical understanding of physiology (Liu et al., 2015), within populations through point measurements as is the case of our networks (Barajas-Martínez et al., 2020), but also between individuals as shared characteristics to generate phenotypic clusters (Mihaicuta et al., 2017). While most networks in biomedical sciences are constructed of nodes and links of the same nature, our network is closer to classical physiological interactions between systems. In human physiology, hormones (and other regulatory systems) exert effects over a wide array of variables regulated variables -blood pressure, electrolytes, protein expression, cellular responses etc., in response to internal needs and to external perturbations. These interactions are very different from usual network approach where only genes, proteins or metabolites are considered, or in the case of neurosciences where different channels of FMRI or EEG are used to construct functional networks. Here we propose a general framework for approaching multivariate datasets of physiological nature that are commonly analyzed through conventional approaches.

Networks are information-rich representations where meaningful characteristics are present in the network topology, layout, clustering, node centrality and edge characteristics. This provides a rich context for interpretation of physiological data. We show that there are robust interactions (links) between physiological variables (nodes), that are preserved between datasets and have very high significances, even for relatively small samples. The analysis of these networks results in similar clustering even when networks are constructed from different datasets. These clusters are not a product of random chance, but are rather built from related variables with underlying mechanisms related to specific functions. Clustering approaches have been used before in the literature, where at least two strategies have been well described. Nodes may be conglomerated through force-directed layouts to generate topological clusters, and through modularity optimization algorithms (Noack, 2009). For an adequate analysis and successful rendering of functional clusters network filtering is critical (Mihaicuta et al., 2017). Here we use the *p*-value, a widely validated strategy to sort significant correlations between variables, to filter the physiological network. Through a modularity optimization algorithm, we clustered physiological variables into functional groups.

### Cluster 1

The variables within this cluster are related to the size of the organism (Supplementary Figures 1, 2). One of the main drivers of body size differences in humans is sexual dimorphism. Males tend to have larger bodies than females with immediate mechanical consequences. For instance, body compartments are larger, including thoracic dimensions, and all the spirometry measurements where anatomic size is important (FVC, FEV1, COadj, AV, kCO, TLC, RV). A larger body also increases some anthropometric characteristics (neck, height, and weight), and bioimpedance measurements (skeletal muscle mass, lean body mass, total body water and intracellular water). Furthermore, a relation between pulmonary function and bioimpedance measurements is present only for lean mass measurements but not for body fat measurements (Park et al., 2012). Sex association with size has also a hormonal context that results in differences in complete cell count and chemistry (HDL, hemoglobin, hematocrit, albumin, and platelets). For the **Project_42** database bilirubin measurements are also present in this cluster, as they are product of degradation of the hemoglobin. Finally, a large lean mass also implies an increased number of metabolites associated with protein and aminoacid replacement (urea, uric acid, and creatinine). These parameters are subject to a hormonal context as they are altered with chronic abuse of androgenic hormones (Navidinia and Ebadi, 2017).

### Cluster 2

This cluster contains nodes related to endocrine regulation. The adipoinsular axis comprehends incretins (Ghrelin, GIP, GLP-1), that signal the food bolus composition, and adipokines, that signal storage state of the adipose tissue (leptin, resistin, visfatin), to tailor the homeostatic response of the pancreatic islet (Kieffer and Habener, 2000). Embedded in this modulation environment, pancreatic beta cells secrete the only hormone that lowers glucose in hyperglycemia (insulin, equimolarly with c peptide), and pancreatic alpha cells secrete a contra-regulatory hormone in hypoglycemia (glucagon). Visceral fat accumulation induces a pro-inflammatory state, resulting in endothelial activation (PAI-1, ICAM-1, VCAM-1, endothelin-1) which allows for circulating immune cells diapedesis (passage from the blood to the tissues) where arrival perpetuates the pro-inflammatory state and produces insulin resistance (Meigs et al., 2004). These closely related functional interactions result in a dense endocrine regulation cluster for the **C22_14** network (Supplementary Figure 3).

### Cluster 3

Erythrocyte characteristics are summarized in clinical settings through red cell indices. Of these, the red cell blood distribution width (RDW) is one of the most recent indices (Salvagno et al., 2015). Mean corpuscular volume (MCV), mean corpuscular hemoglobin concentration (MCHC) and mean corpuscular hemoglobin (MCH) represent average values of volume and hemoglobin content, whereas RDW is a variability-based metric (Sarma, 1990). Together these four parameters allow for classification of anemic disease and provide clinical orientation and are found clustered in the **C22_14** network (Supplementary Figure 4).

### Cluster 4

White blood cells (leukocytes) are the cellular component of the immune system that flows through the blood. These cell types are orchestrated in several immune responses but have been more or less well categorized in specialized functions and are clustered in the **C22_14** network (Supplementary Figure 5). For instance, neutrophils and monocytes are part of the innate immune response. An “always ready” system for immediate deterrence of infectious pathogens. On the other hand, lymphocytes, eosinophils and basophils participate in the adaptative immune response. A tailored cellular response to effectively resolve infectious processes that have overcome responses of the innate immune system. For the **Project_42** network, Cluster 3 physiological variables are included in this cluster, along with platelets and mean platelet volume (MPV), comprising al cellular components in the blood (Supplementary Figure 6). These physiological parameters have been related to cardiovascular risk, placing them in the context of a wider set of interactions beyond infection response (Hansen et al., 1990; Madjid and Fatemi, 2013). Nitric oxide in exhaled breath is present in this cluster and relates to eosinophils, as expected since eosinophils are a major source of NO in asthma (MacPherson et al., 2001).

### Cluster 5

Lipids present in blood, and their associated carrier proteins, are classical biomarkers of cardiovascular risk and were clustered in both networks (Supplementary Figures 7, 8). Triglycerides and total cholesterol were first identified. Later, cholesterol was separated into fractions according to weight, unveiling a transport system composed of lipoproteins that carry lipids from their storage depots to the cells, VLDL, IDL, and LDL, and lipoproteins that carry lipids from the cells into the storage depots, HDL (Ito and Ito, 2020). In epidemiological studies HDL levels have shown to be protective against cardiovascular disease, while LDL levels represent a risk factor. ApoA and ApoB, the protein envelopes that carry the lipids in these fractions, showed better predictive results. However, upon increasing knowledge of the physiopathology of vascular disease new biomarkers have been assessed such as lipoperoxidation products, serum phospholipids and oxidized LDL (Ngoc-Anh, 2009). As **Project_42** has less lipidic variables this cluster comprises only LDL, HDL and cholesterol.

### Cluster 6

Visceral adiposity is the main driver of metabolic disease. It has been measured through several proxies including body weight and waist circumference either as individual measurements or as composed indices (BMI, height/waist ratio), and by indirect measurement using bioimpedance (total body fat, visceral fat, fat free mass, body fat, superficial body fat). These variables are clustered in both networks (Supplementary Figures 9, 10). Over time, excess of visceral adipose tissue triggers a low-grade chronic pro-inflammatory state, as revealed by high sensitivity but low specificity C reactive protein, an acute phase pentraxin produced in the liver (Pettersson-Pablo et al., 2019). Increased visceral adiposity is also related to high blood pressure (systolic and diastolic blood pressure) through several mechanisms even in young adulthood (Takeoka et al., 2016). The variables in this cluster share the property of being very stable over time. Body fat deposits, resting blood pressure and glucose levels are rather stable variables that vary only over very long periods of time. For **Project_42** anthropometric measurements, such as hip and arm circumference, as well as all skinfolds of plicometry, are located in this cluster (Supplementary Figure 10).

### Cluster 7

Carbon monoxide cluster. There is a final cluster that comprises the relation between exhaled carbon monoxide (CO) and hemoglobin irreversibly bound to CO in blood (COHb) (Wald et al., 1981). As none of the participants in the **C22_14** dataset were smokers this cluster is relatively well isolated from the network (Supplementary Figure 11). Nonetheless, breath profiles including CO and NO have been proposed for monitorization of whole body states (Maiti et al., 2019).

The inter-cluster correlations manifest the integration between these different functional systems within the organism, as well as some physiological variables placed as intermediaries in the network between clusters (Figure 9). As force-directed algorithms may work as energy-models (Noack, 2009), location of the nodes within the layout is also informative of the role nodes may have in the functional cluster, either deep inside or in the periphery (Supplementary Figures 12, 13). The robust agreement between the present network approach and medical knowledge invites us to extend network analysis to physiological phenomena. It has been suggested previously that topology characteristics of a network have functional implications that are not observable by reductionist approaches (Ivanov and Bartsch, 2014). A network framework for physiological understanding may facilitate immediate comprehension of distant interactions and emergent properties of living systems.

Living systems are neither completely random nor fully ordered. This property has been noted at multiple levels of observation. For example, from a time-series perspective, the analysis of continuous heart rate data reveals that balance between robustness and adaptability of the cardiovascular system is an important biomarker of health (Rivera et al., 2018). For networks, this is the essence of a complex topology, such as small world or scale-free, since they feature patterns of connection between their elements that are neither purely regular nor purely random. In Figure 10 we show that both networks have a scale-free topology (with some degree of both random and orderly structure). In summary, the complex behavior of living systems in time series appears to be reflected also in network physiology.

The limitation of our study is the small size of our datasets, nevertheless our methodology combines parameters that are not usually related to build a physiological network. Moreover, the physiological network constructed is robust and similar for both datasets.

## Conclusion

Textbooks on basic physiology present homeostatic regulation of cardiovascular, respiratory, metabolic and other subsystems as if they were independent mechanisms coordinating the dynamics of closely related variables in order to create a stable local environment that can be studied from the perspective of separate medical disciplines (Ganong, 1969; Hall, 2011). This is of course a coarse approximation because it is implicit that the different subsystems must interact in order to assure a system-wide homeostatic state, remaining outside the scope of the reductionist approach to physiology. Systems biology, on the other hand, suggests that systemic homeostasis “emerges” from an underlying network and interactions between variables that span the whole system (Goldstein, 2019). Figures 7–9 show 6 distinct clusters, where the intra-cluster interactions between related variables may well represent the textbook examples of local subsystem homeostasis, whereas the inter-cluster interactions between very distinct variables most probably convey new and unexplored information of how homeostasis is established at the system level in the optimal conditions of youth and health, and how the loss of homeostasis arises with aging and/or disease.

## Data Availability Statement

The raw data supporting the conclusions of this article will be made available by the authors, without undue reservation.

## Ethics Statement

The studies involving human participants were reviewed and approved by The Ethics Committee of the “Instituto Nacional de Enfermedades Respiratorias” (INER) approved the procedures and protocols for this study under project C22_14. The Ethics Committee of the Facultad de Medicina of the Universidad Nacional Autónoma de México (UNAM) approved the procedures and protocols for this study under project FM/DI/023/2014. The patients/participants provided their written informed consent to participate in this study.

## Author Contributions

AB-M, EI-C, and ALR performed data analysis, network modeling, and wrote this work. CRS did the conceptualization of the project for dataset of Project_42 and funding acquisition. AB-M participated in methodology and data collection. MPS-V did the conceptualization of the project for dataset C22_14, funding acquisition, and resources. OGA-A and YD-G did the data curation. OGA-A and MPS-V did the investigation. CV-D, RG-T, KB, OGA-A, and MPS-V did the methodology. ALR, EI-C, RF, VMG, OAL, AOM-G, and AF participated in the complexity interpretation and analysis of the results. MANM, DAMO, CEL-C, IC-B, PA-V, and CAA-S participated in data analysis and medical interpretation in this work. All authors contributed with the manuscript revision, read and approved the submitted version.

## Conflict of Interest

The authors declare that the research was conducted in the absence of any commercial or financial relationships that could be construed as a potential conflict of interest.
