# Supplementary material for: Physiological Network From Anthropometric and Blood Test Biomarkers
Source: Front Physiol. 2021 Jan 12;11:612598. doi: 10.3389/fphys.2020.612598 (PMC7835885; doi:10.3389/fphys.2020.612598)
Supplement: Supplementary file 1 [file Data_Sheet_1.PDF]

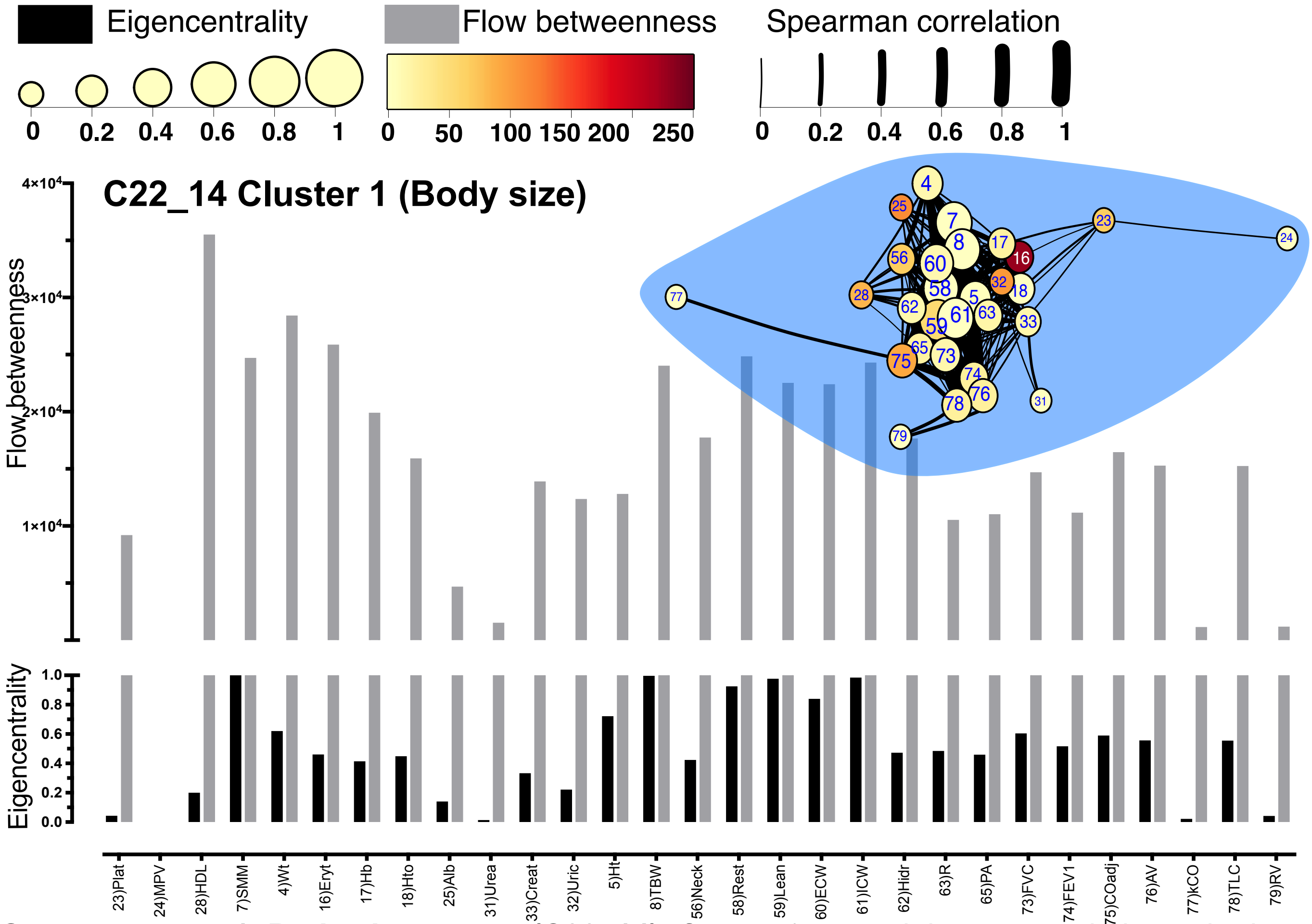

Supplementary 1. Body size cluster (C22_14). Cluster 1 network is presented above the bar graph. The figure legend placed on top indicates the bar color assigned to each centrality and the network representation of eigencentrality, flow betweenness and Spearman correlation.

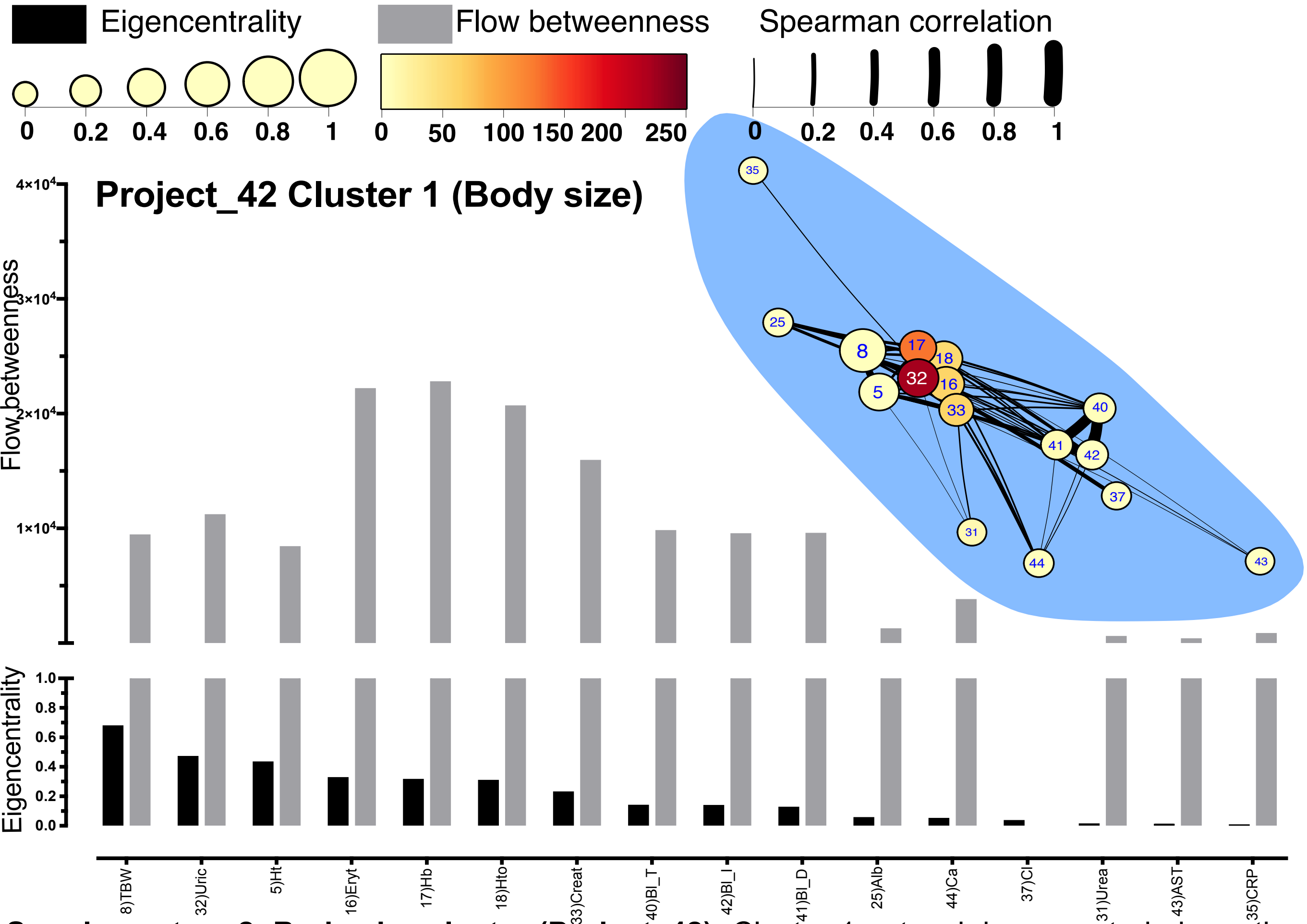

Supplementary 2. Body size cluster (Project_42). Cluster 1 network is presented above the bar graph. The figure legend placed on top indicates the bar color assigned to each centrality and the network representation of eigencentrality, flow betweenness and Spearman correlation.

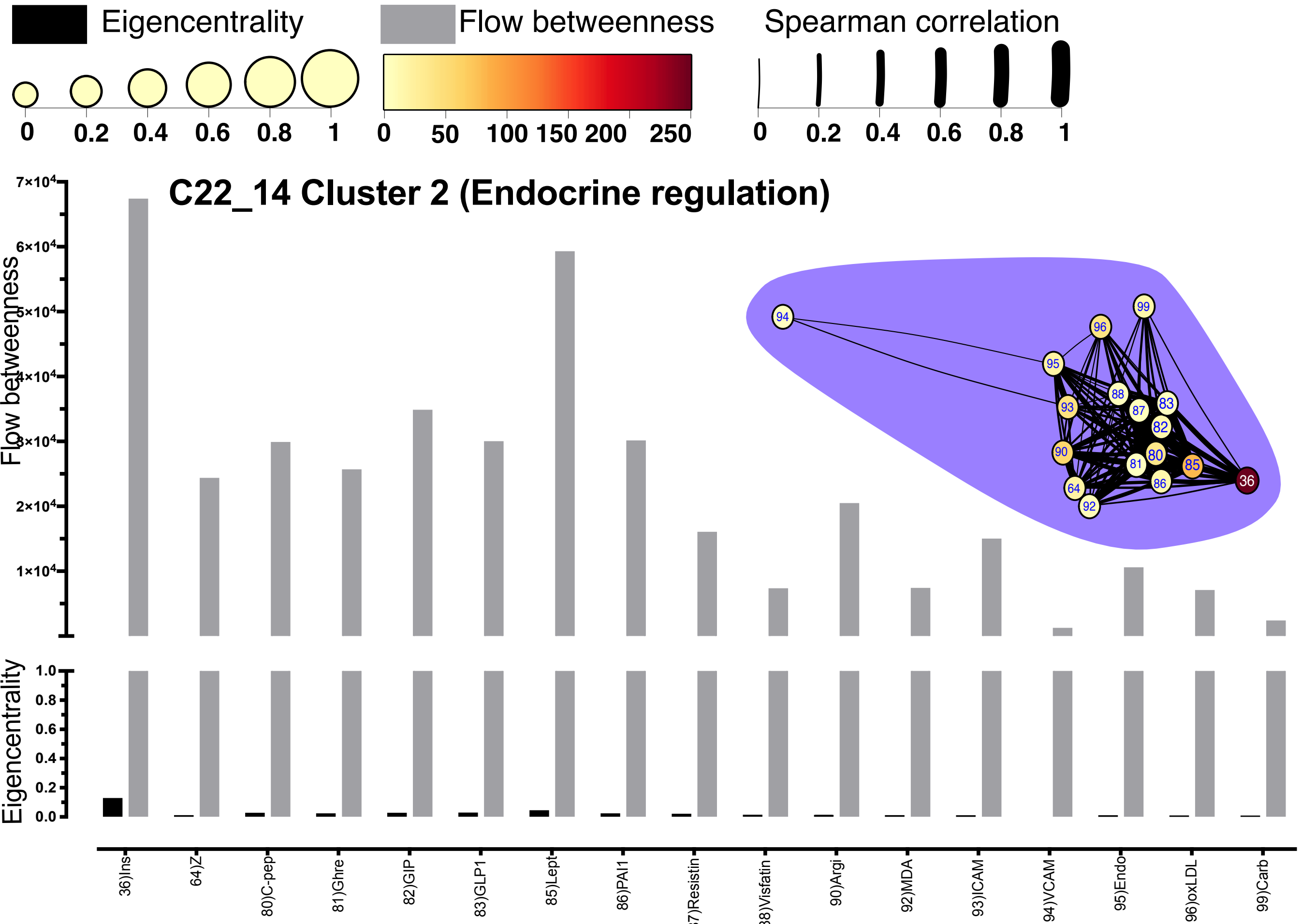

Supplementary 3. Endocrine regulation cluster (C22_14). Cluster 2 network is presented above the bar graph. The figure legend on top indicates the bar color assigned to each centrality and the network representation of eigencentrality, flow betweenness and Spearman correlation.

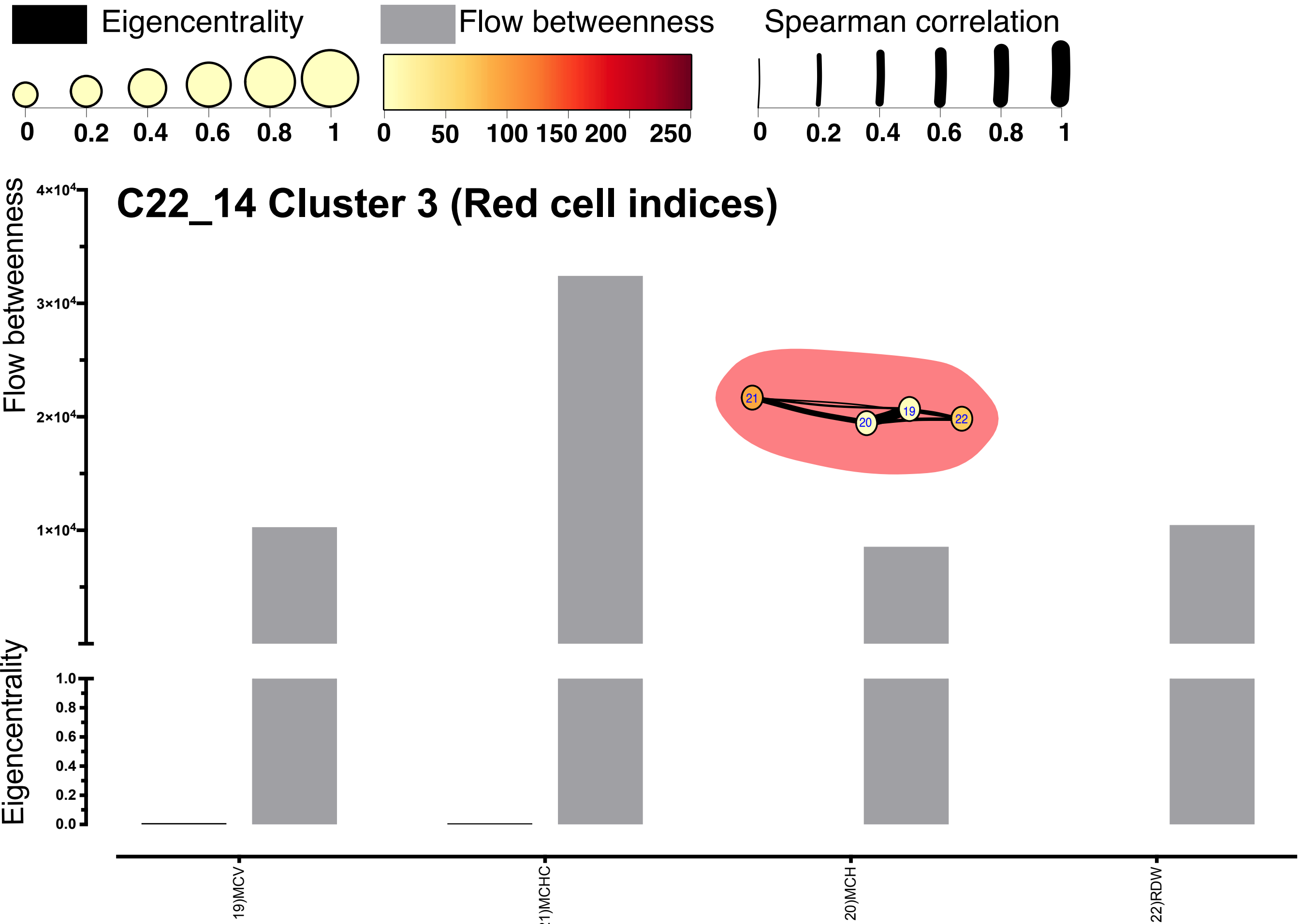

Supplementary 4. Red cell indices (C22_14). Cluster 3 network is presented above the bar graph. The figure legend placed on top indicates the bar color assigned to each centrality and the network representation of eigencentrality, flow betweenness and Spearman correlation.

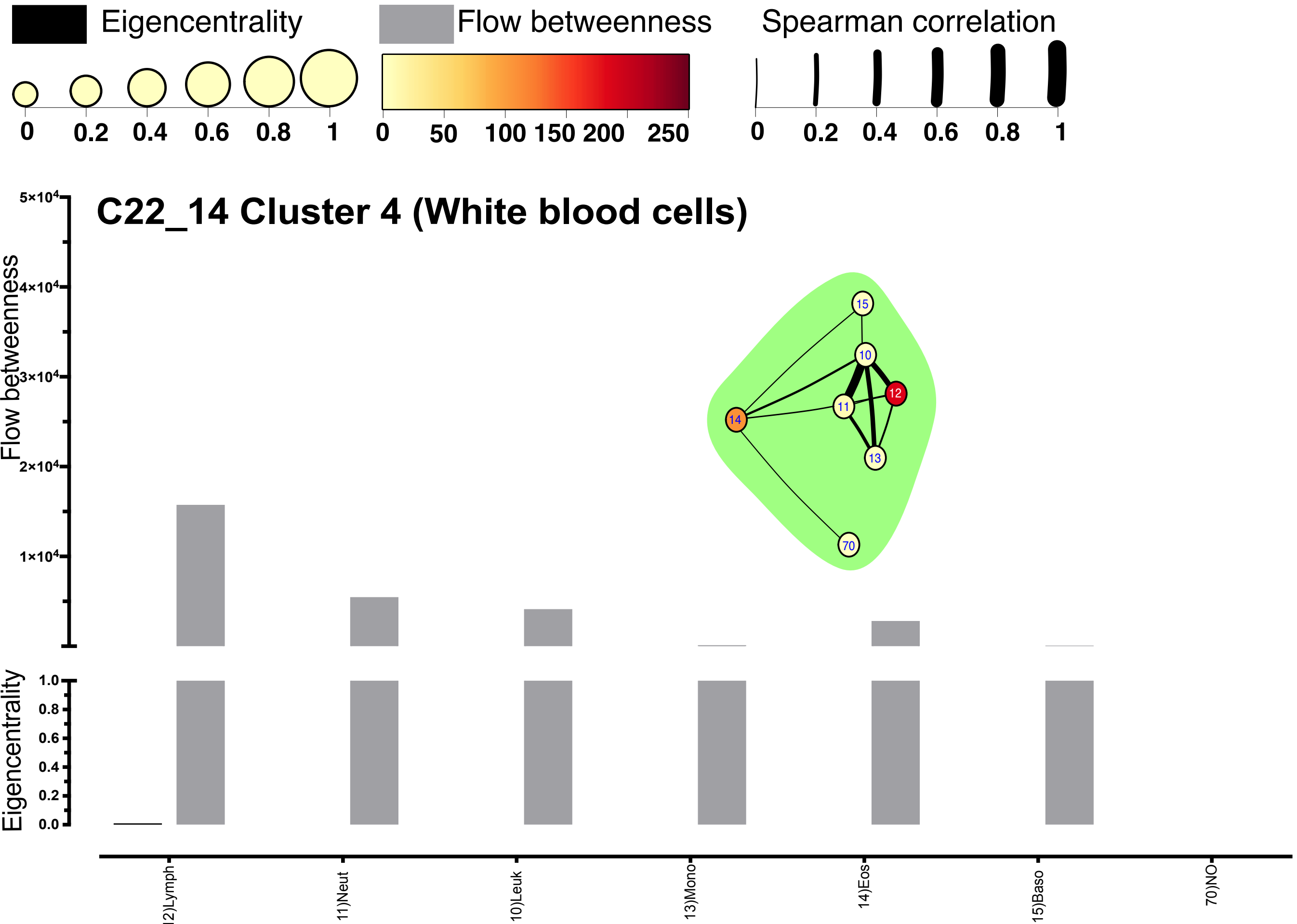

Supplementary 5. White blood cells (C22_14). Cluster 4 network is presented above the bar graph. The figure legend on top indicates the bar color assigned to each centrality and the network representation of eigencentrality, flow betweenness and Spearman correlation.

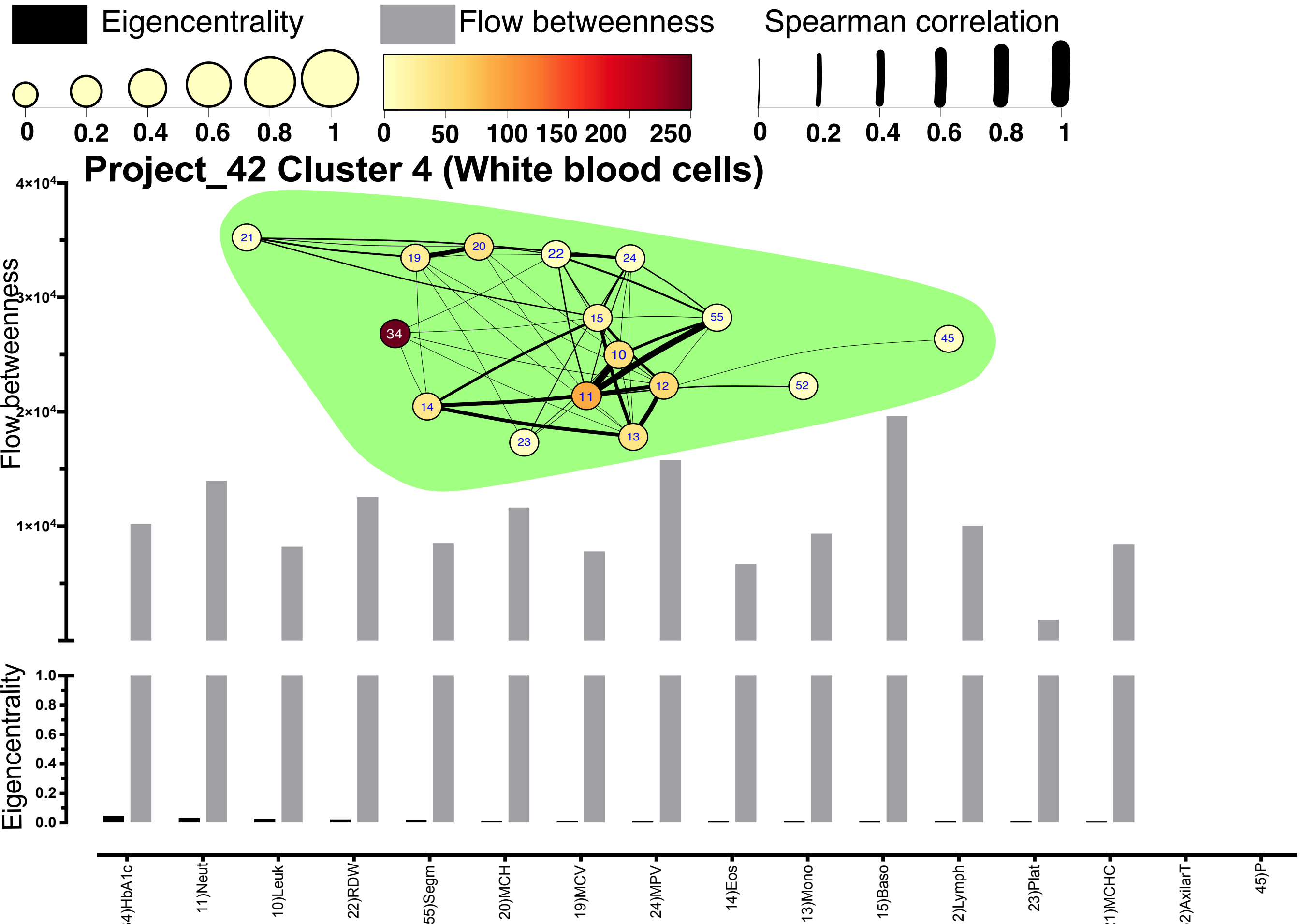

Supplementary 6. White blood cells cluster (Project_42). Cluster 4 network is presented above the bar graph. The figure legend on top indicates the bar color assigned to each centrality and the network representation of eigencentrality, flow betweenness and Spearman correlation.

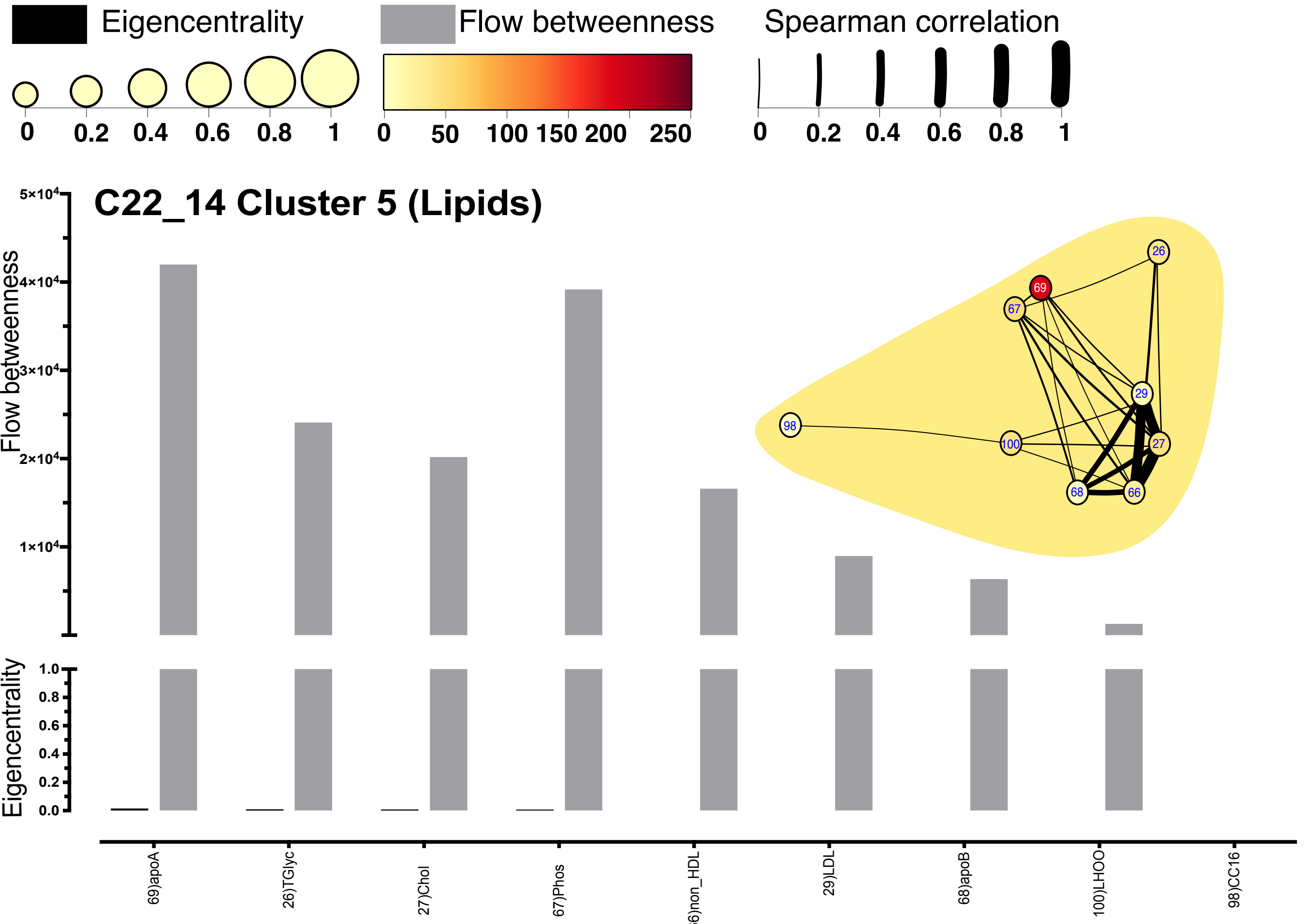

Supplementary 7. Lipids cluster (C22_14). Cluster 5 network is presented above the bar graph. The figure legend on top indicates the bar color assigned to each centrality and the network representation of eigencentrality, flow betweenness and Spearman correlation.

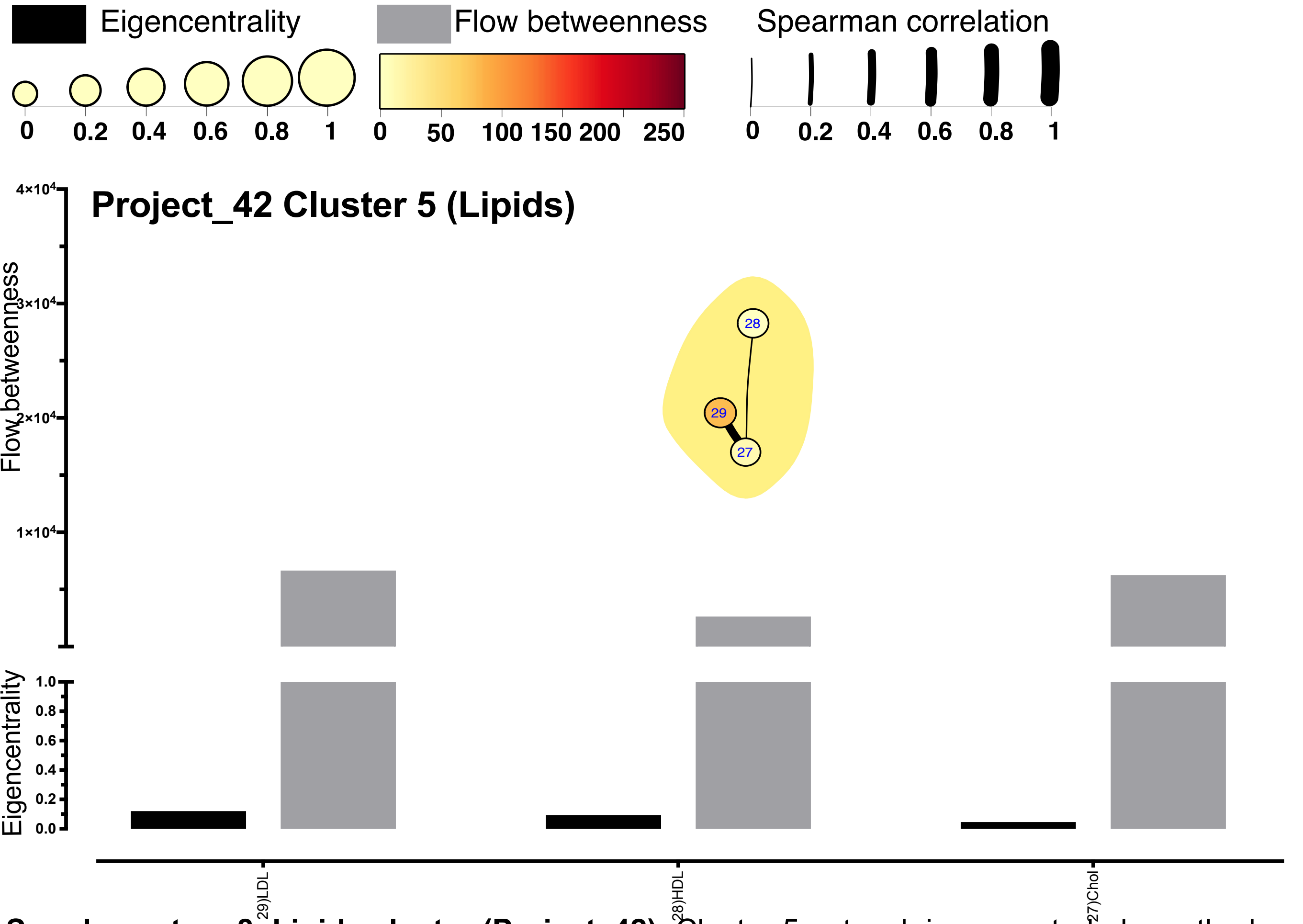

Supplementary 8. Lipids cluster (Project_42). Cluster 5 network is presented above the bar graph. The figure legend on top indicates the bar color assigned to each centrality and the network representation of eigencentrality, flow betweenness and Spearman correlation.

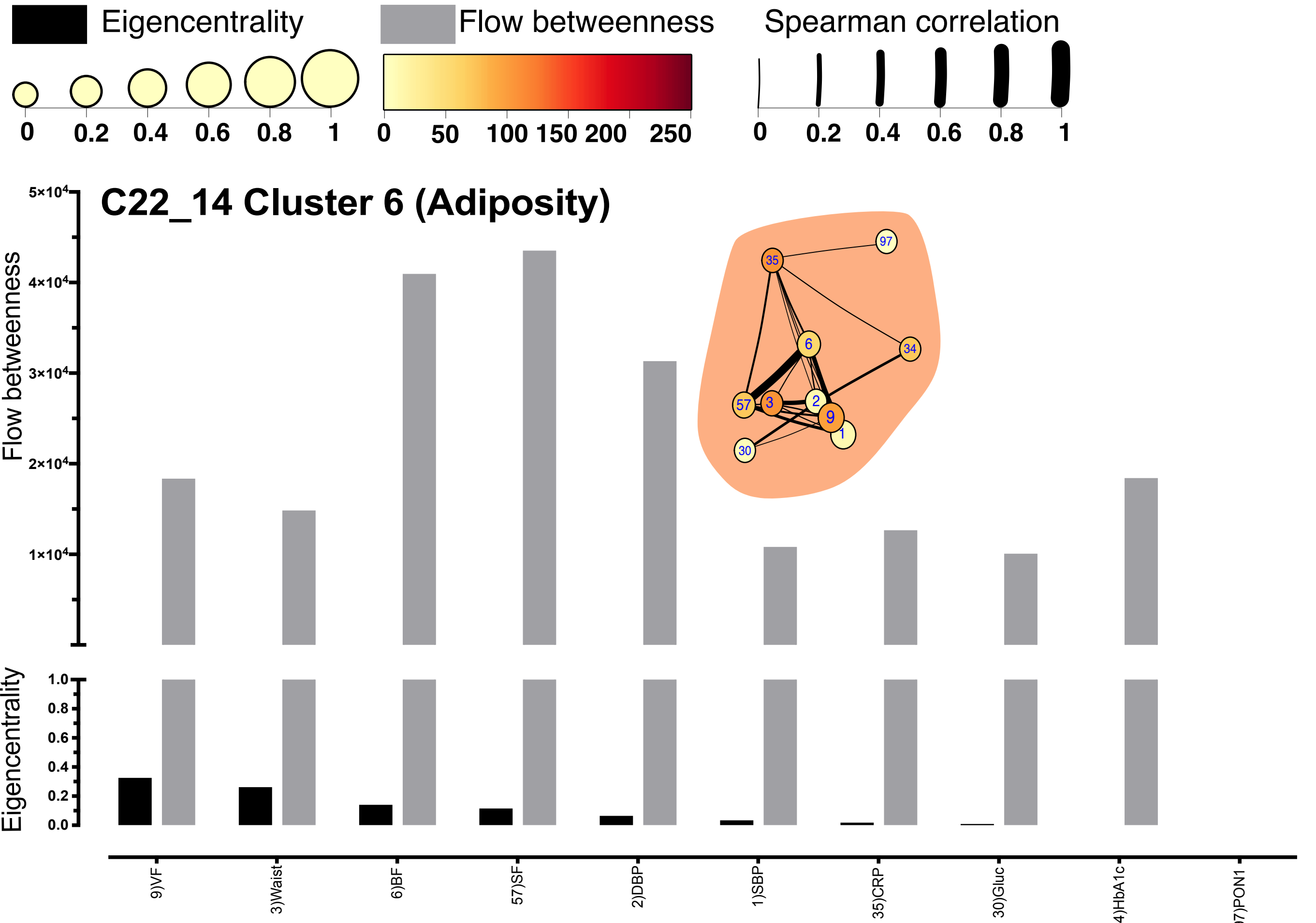

Supplementary 9. Adiposity cluster (C22_14). Cluster 6 network is presented above the bar graph. The figure legend on top indicates the bar color assigned to each centrality and the network representation of eigencentrality, flow betweenness and Spearman correlation.

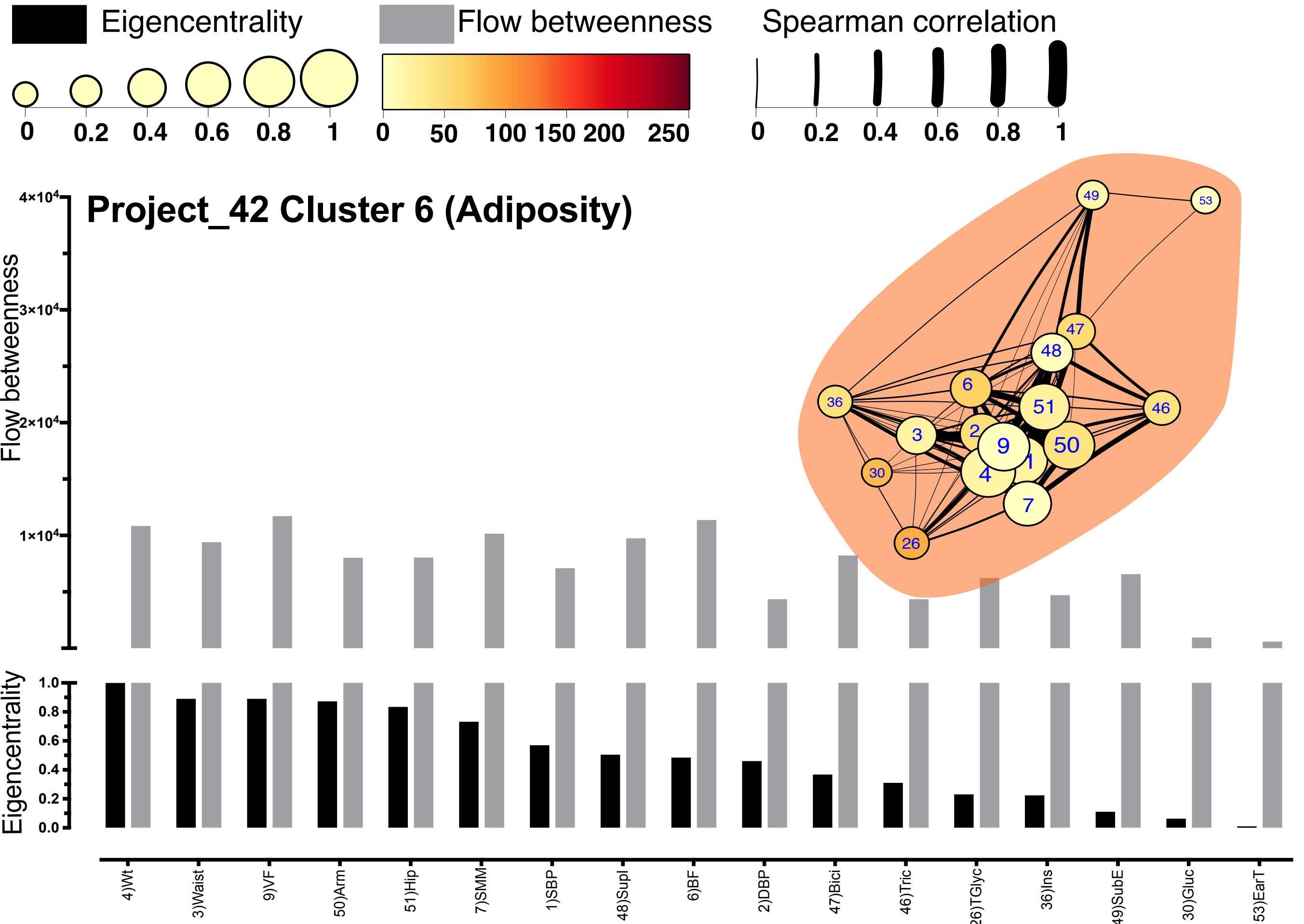

Supplementary 10. Adiposity cluster (Project_42). Cluster 6 network is presented above the bar graph. The figure legend on top indicates the bar color assigned to each centrality and the network representation of eigencentrality, flow betweenness and Spearman correlation.

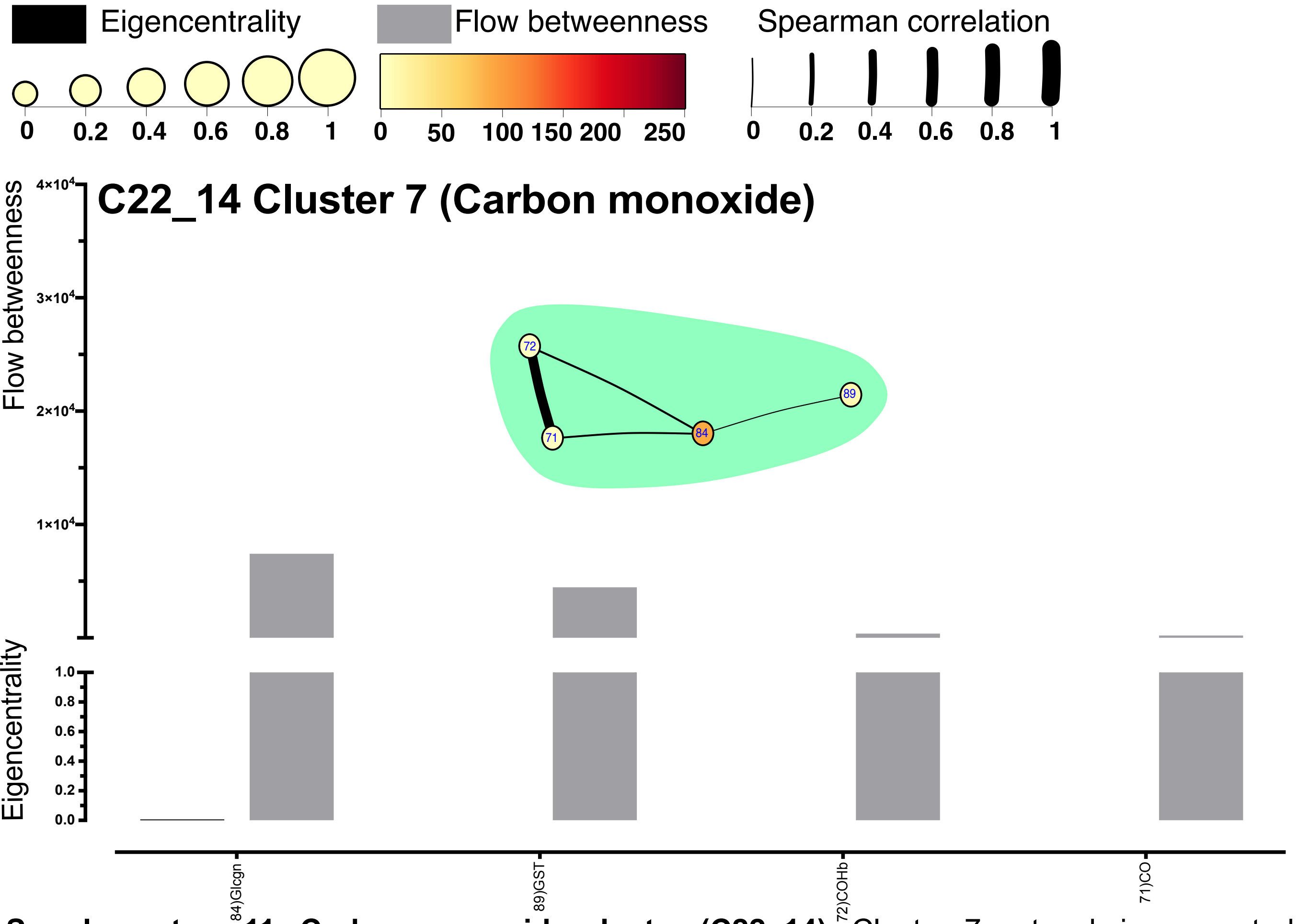

Supplementary 11. Carbon monoxide cluster (C22_14). Cluster 7 network is presented above the bar graph. The figure legend on top indicates the bar color assigned to each centrality and the network representation of eigencentrality, flow betweenness and Spearman correlation.

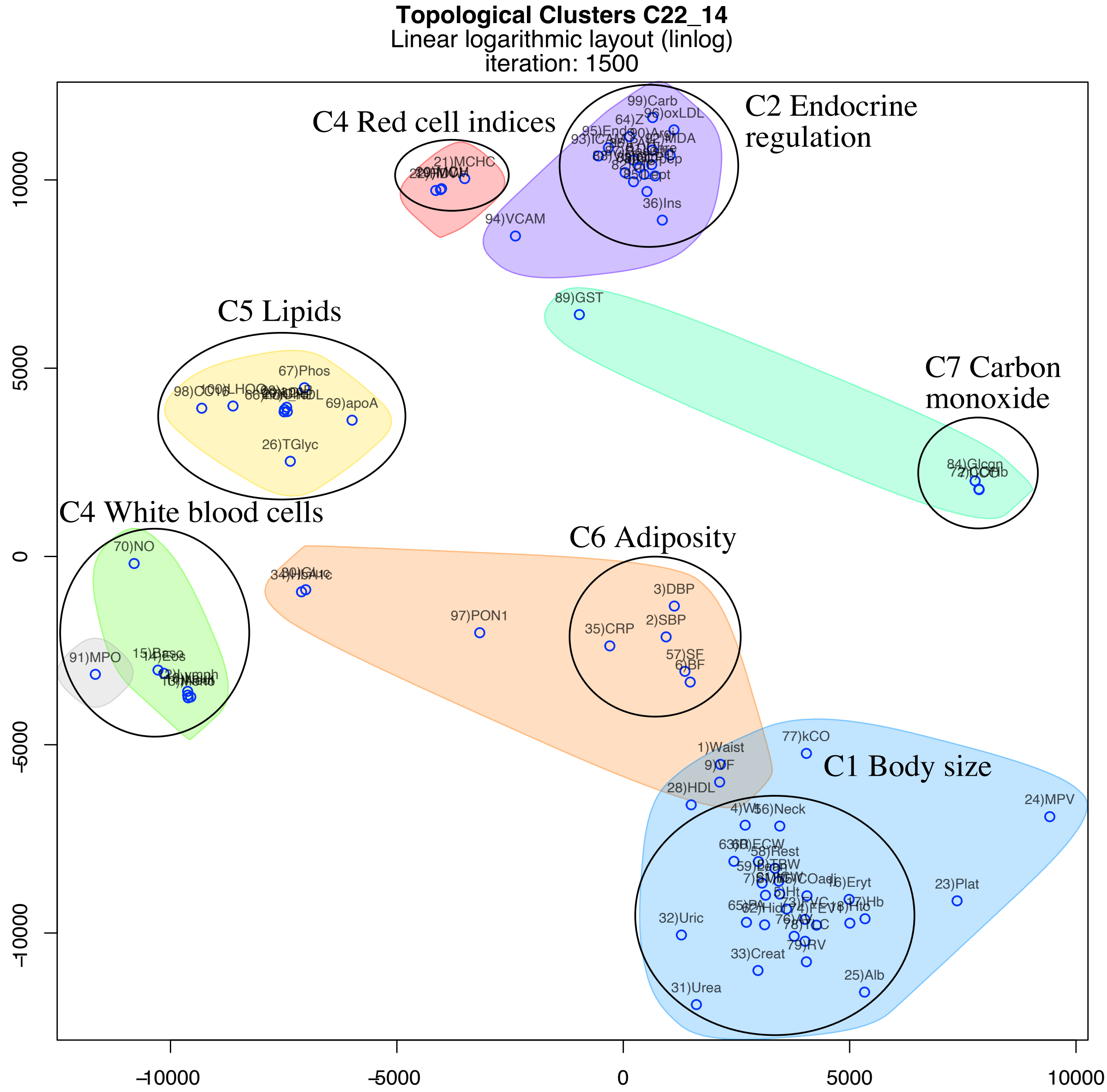

Supplementary 12. Topological clusters (C22_14). Topological clusters that appear in the Linlog model are labelled inside the black ellipses, clusters from Louvain algorithm appear as color shadowed areas.

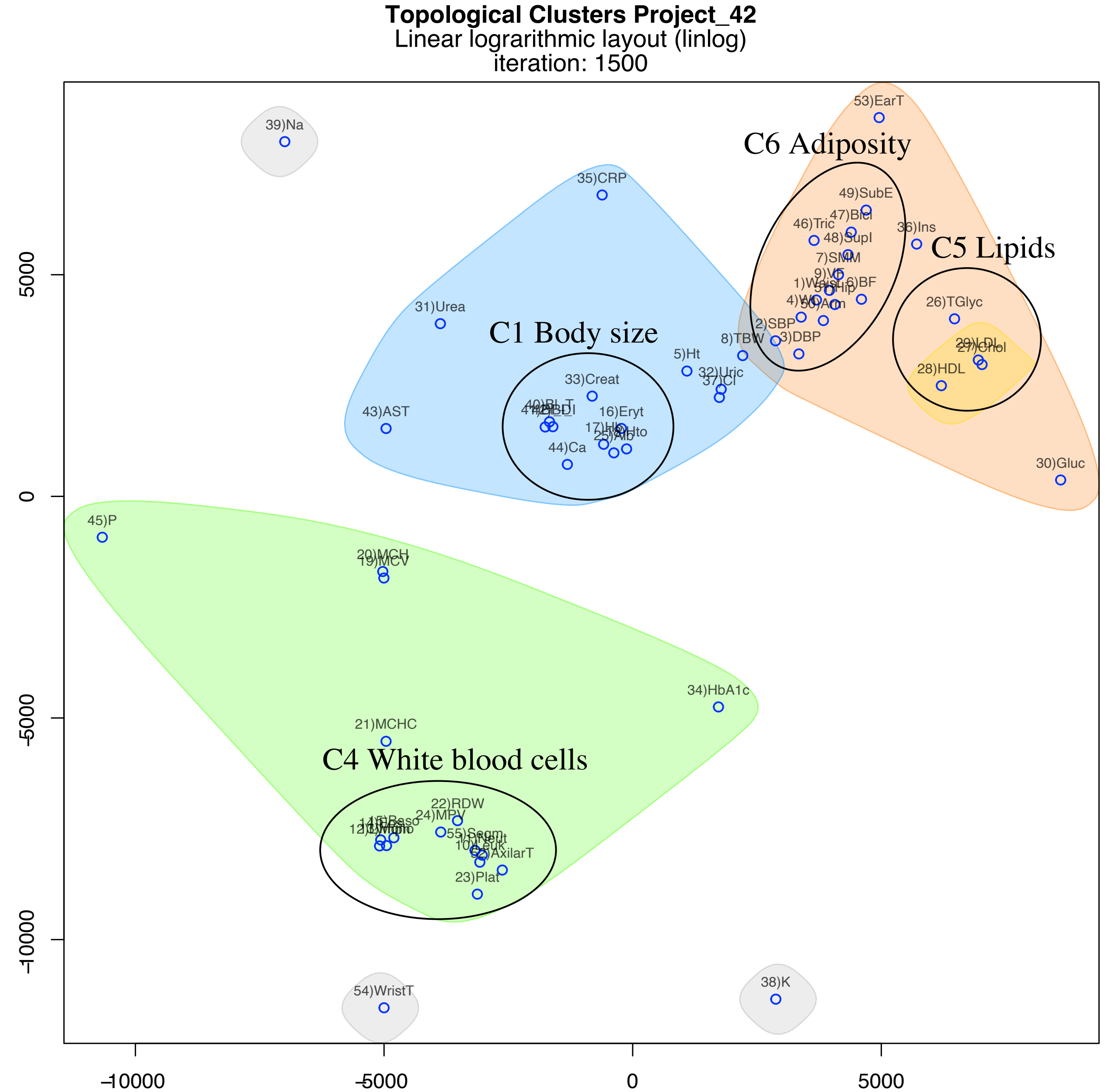

Supplementary 13. Topological clusters (Project_42). Topological clusters that appear in the Linlog model are labelled inside the black ellipses, clusters from Louvain algorithm appear as color shadowed areas.

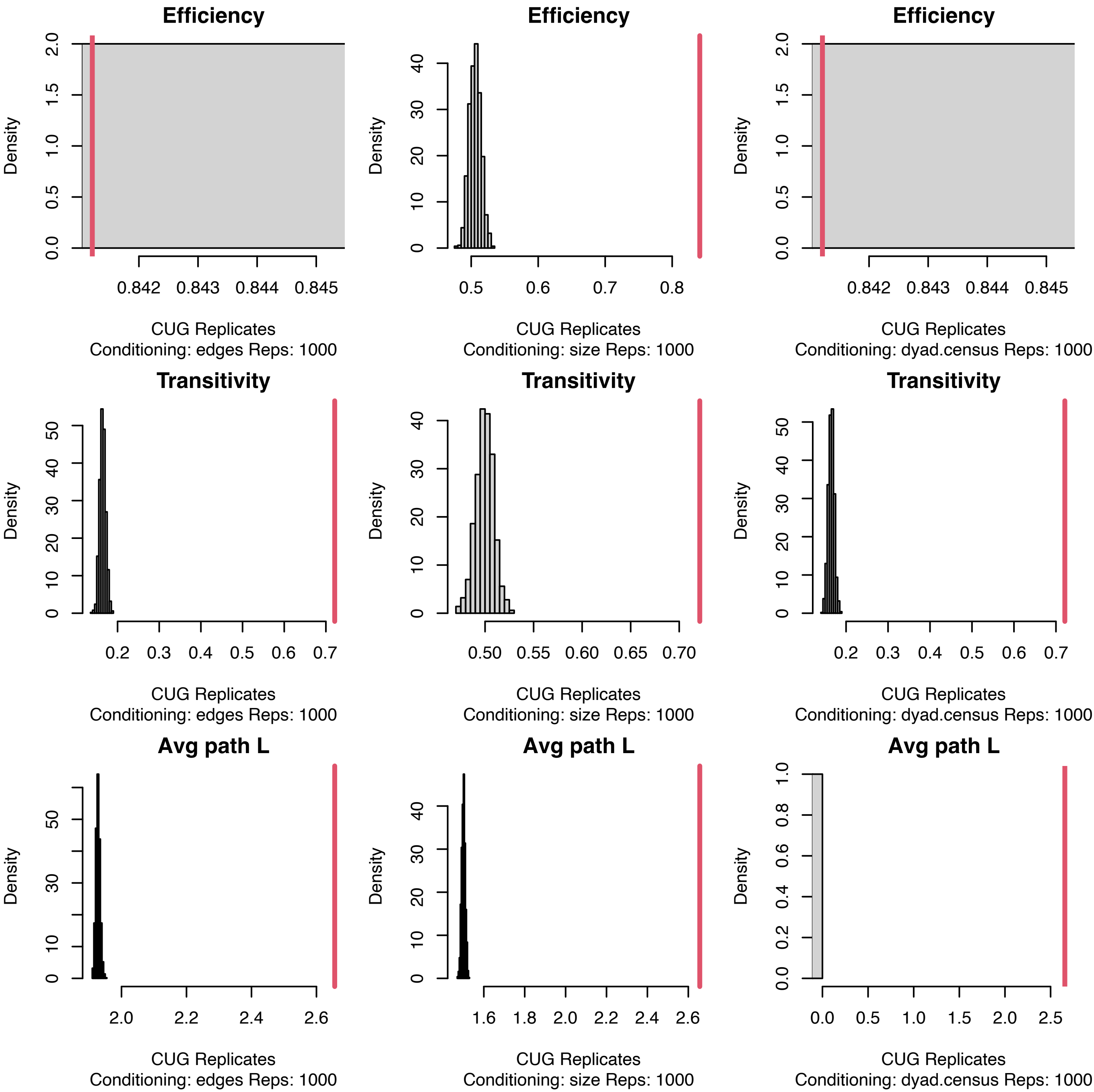

Supplementary 14. Conditionally uniform graph tests (C22_14). First column shows the distribution of CUG fo edges, second column for size and third column for dyad census. Rows show first efficiency, second transitivity and third average path length. The observed value is presented as a red line.

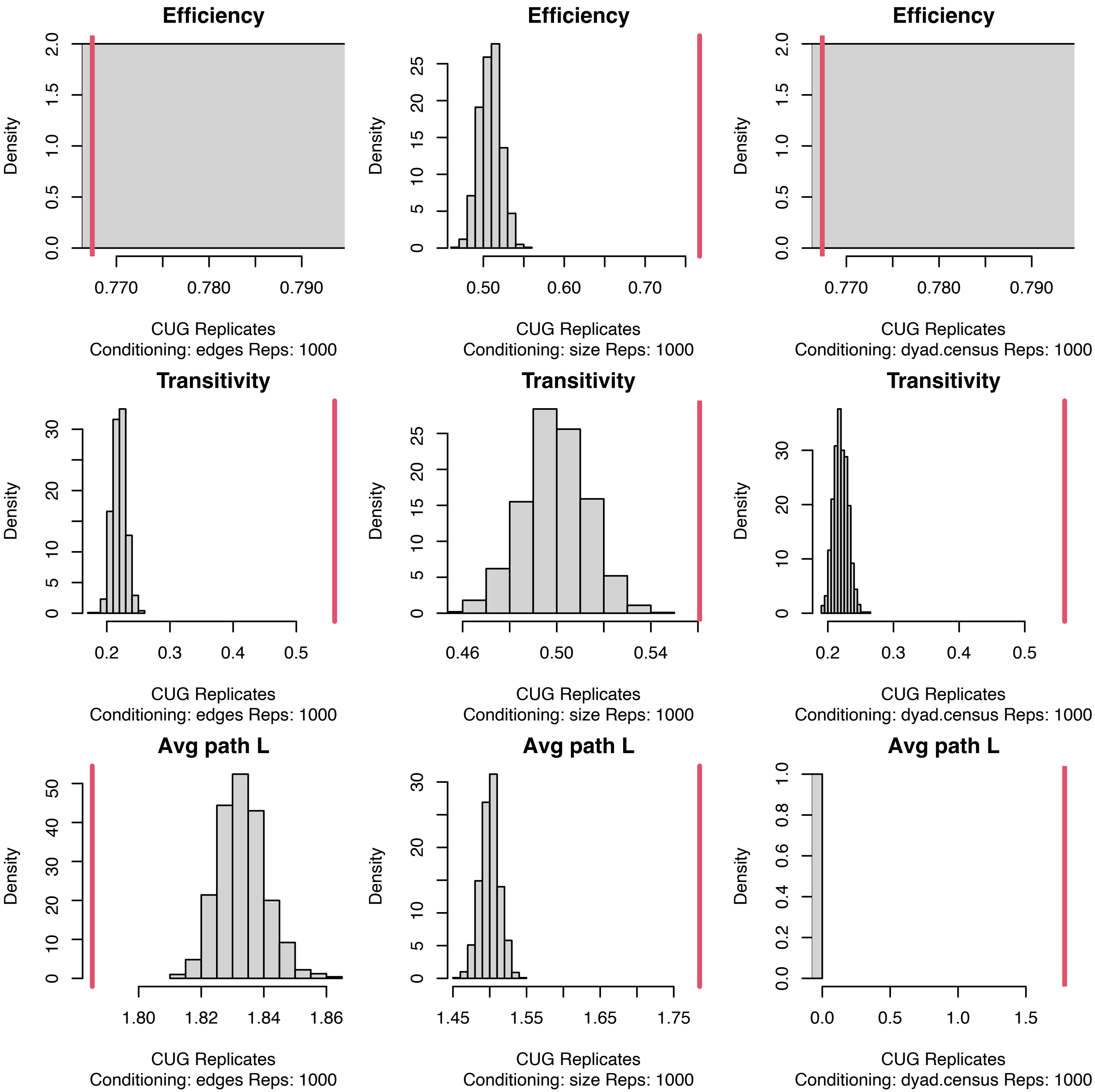

Supplementary 15. Conditionally uniform graph tests (Project_42). First column shows the distribution of CUG fo edges, second column for size and third column for dyad census. Rows show first efficiency, second transitivity and third average path length. The observed value is presented as a red line.
